# Nonuniform Distance Decay of Conductance in a Series of Multiheme Cytochrome Bioelectronic Junctions

**DOI:** 10.1021/acs.jpclett.6c01449

**Published:** 2026-07-31

**Authors:** A. M. Pethő, Zdenek Futera, Jochen Blumberger

**Affiliations:** † Department of Physics and Astronomy and Thomas Young Centre, 4919 University College London, London WC1E 6BT, United Kingdom; ‡ Faculty of Science, 48271 University of South Bohemia, 370 05 Ceske Budejovice, Czech Republic

## Abstract

Multiheme cytochromes have emerged as a promising new class of bioelectronic materials for potential use in soft and biocompatible electronics. To aid these developments it is essential to understand how conductance decays with increasing size of multiheme proteins and which parts of the proteins govern their high conductance. Here we present a systematic investigation of six native multiheme cytochromes forming electronic junctions of increasing size. We find that the computed conductances are best described by a protein-specific intercept model with a single exponential distance decay constant of β = 1.8 nm^–1^ shared across all six proteins and an increasing intercept with protein size. Moreover, our calculations suggest that conductance for larger multiheme cytochromes tends to be less sensitive to thermal protein fluctuations than for smaller proteins. Both observations can be explained by the higher density of protein electronic states with increasing protein size. Fe ions appear to be unimportant for electronic conduction and the contribution of heme-cofactors is relatively small compared to their essential role for electron transfer in native biological environment. Our calculations suggest that it is mainly the protein frame and the contacts between amino acids and electrodes that govern electronic conductance in the series of multiheme cytochromes investigated.

## Graphical abstract

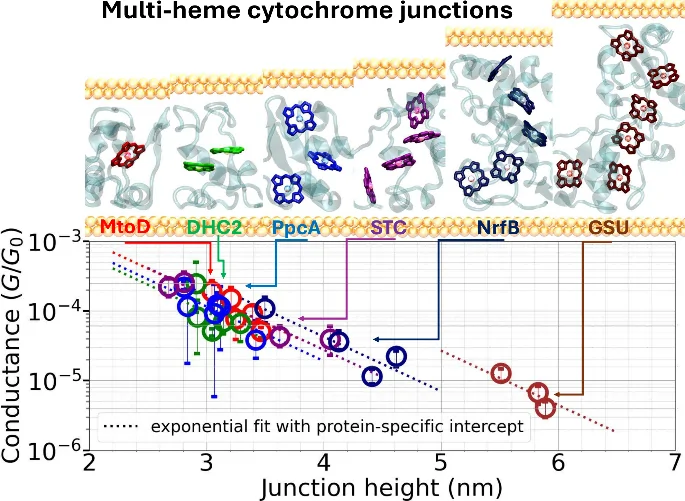

Biomolecular electronics aims to exploit the exquisite ability of biomolecules to mediate electronic current in man-made electronic circuits. Indeed, the integration of proteins as building blocks in electronic devices is a rapidly growing field that opens up exciting opportunities for applications in biosensing, biotransistors, biopowering and biocompatible implantable electronics.^1–7^ Many aspects of the contemporary Si-based microelectronics would be surpassed by bioelectronics technology: low power consumption, biocompatibility and ready integration in flexible electronics. However, on a fundamental level, how proteins conduct electronic currents when taken away from their natural cellular habitats and placed between two metal electrodes is still a matter of intense discussions,^8–24^ by contrast to molecular electron transfer (ET) in biomolecules, the basis of which is now largely established.^25–32^

Pioneering fundamental research on charge transport in protein monolayer solid-state devices^10,13,14,33–36^ and in single-protein scanning tunnelling microscopy junctions^15,37–46^ have demonstrated remarkably efficient long-range electronic transport (ETp), also termed electronic conduction, in some cases rivalling those of organic semiconductors, i.e. molecules with conjugated molecular backbones.^47^ However, some experimental results have been particularly unexpected. First, the electronic current across a range of different proteins has been reported to show a very weak or virtually zero temperature dependence.^10,13,14^ This is in striking contrast to molecular ET in biology which (apart from photoexcited ET) are all thermally activated.^25–27,48^ Second, the distance decay of electronic conduction was found to be exponential with exponential decay constants (β = 1–2 nm^–1^ or less)^14,15^ that are about an order of magnitude smaller than for molecular ET in biomolecules (β = 10–15 nm^–1^).^49,50^ Third, for bacteriorhodopsine temperature-independent conduction with a very shallow exponential distance decay has been reported over no less than 16 nm.^14^

Absence of temperature dependence and exponential distance decay are usually interpreted in terms of a coherent transport process where the charge carrier tunnels from one electrode across the protein to the other electrode in a single step without exchanging energy with the environment. Indeed, our previous modeling studies on the small tetra-heme cytochrome (STC) provided substantial indirect and direct support for an off-resonant coherent tunneling scenario.^10–12,15^ First, an incoherent charge hopping process could be ruled out: hopping models could not fit simultaneously the experimental *I*-*V* and *I*-*T* curves.^11^ Moreover, the computed hopping currents were >10 orders of magnitude lower than the experimental currents due to the large energy barrier for charge injection from the electrode into the protein,^12^ as confirmed by experimental photoemission spectroscopy.^11^ By contrast, analytic coherent tunnelling models (Simmons and Landauer) could fit experimental *I*-*V* and *I*-*T* curves near perfectly,^11^ and in further support for coherent tunneling, the current–voltage response computed using the Landauer-Büttiker formalism with electronic parameters from DFT+Σ calculations gave conductances and exponential distance decay constants in close agreement with experiment.^11,15^

Yet, many questions in single protein conductance remain open. For instance, how universal is the distance decay of electronic conductance for proteins within the same protein family? Can they be described by the same exponential distance dependence, as is the case for molecular electron transfer in biology? How can it be that the finite temperature fluctuations of proteins support a coherent electron transport scenario over such long distances? What is the role of the transition metal(s) and cofactors, if present, and what is the role of the protein frame in mediating electronic current? “First-principles”-type DFT+Σ ^51–53^ electronic structure calculations on protein junctions are arguably one of the best methods to address these questions systematically but they are computationally expensive and were limited in the past to one or two relatively small proteins (like STC).^11,12,15^ This has now changed due to improvements in code and computing performance making an investigation of these questions now very timely. Thus, in the following we present a systematic computational study of protein electronic conductance considering six different multiheme cytochromes of increasing size binding 1, 2, 3, 4, 5, and 6 heme cofactors and forming junctions of increasing width (Figure 1).

**Figure 1:**
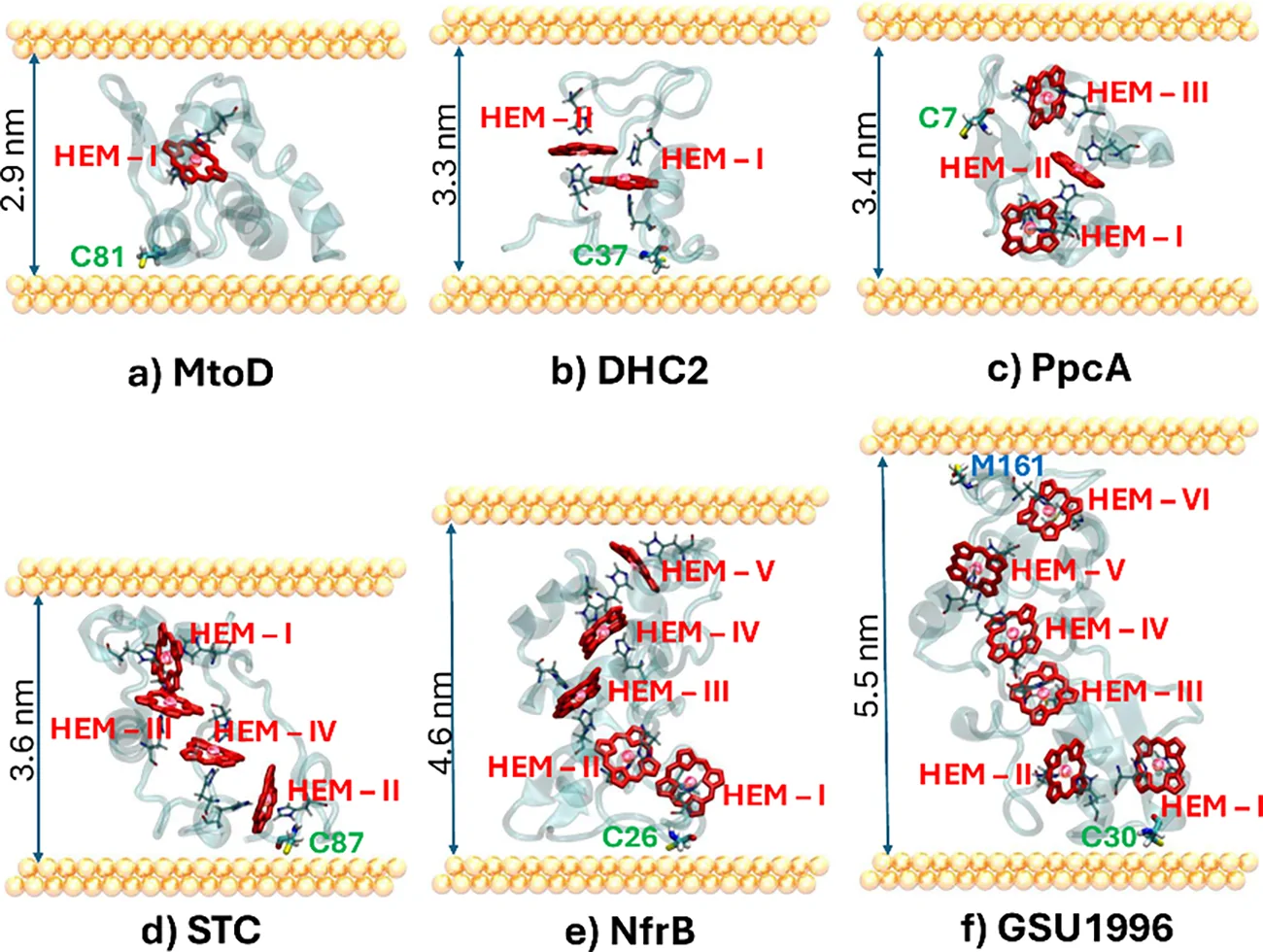
Structure of 6 multiheme cytochrome junctions studied in this work. Monoheme protein MtoD (a), diheme protein DHC2 (b) triheme protein PpcA (c), tetra-heme protein STC (d), penta-heme protein NrfB (e) and hexa-heme protein GSU (f). For each protein an orientation was chosen where a cysteine residue is chemisorbed on the bottom electrode (except in PpcA) and the heme chain is approximately pointing along the surface normal. The secondary structure of the protein is depicted in green, the Fe-heme cofactors in red and the Au(111) electrodes in yellow. Cysteine (C) and methionine residues (M) interacting with the Au electrodes are indicated in stick representation. See main text and Supporting Information (SI) for details regarding the preparation of the structures.

Au­(111)-protein-Au­(111) junctions were generated for the mono-, di-, tri-, penta-, and hexaheme proteins MtoD, DHC2, PpcA, NrfB, and GSU in vacuum starting from the experimentally resolved crystal structures. Junctions for the tetra-heme protein STC were taken from previous work.^12^ These proteins were chosen because (i) they are representative of bacterial multiheme cytochromes that feature an array of tightly bound bis-His ligated heme cofactors that are arranged in stacked and T-shaped orientation (except for GSU where two heme cofactors are ligated by His/Met) and (ii) they are still small enough to be treated at a full explicit electronic structure level when incorporated in a junction (<200 amino acid residues). Vacuum conditions were chosen in our calculations similar to those in the conductance measurements on MHC monolayers in ref.^10^ Since in experiments one often introduces Cys and/or Met residues at opposing ends of the protein to ensure good electronic contact with the electrode (owing to the high affinity of sulfur-containing amino acids with Au), the following point mutations were made: MtoD: S81C and L45M, DHC2: T37C, PpcA: K7C, NrfB: A26C, GSU: A30C (STC: S87C). The mutated proteins were first equilibrated to 300 K in a vacuum using MD simulations followed by slow adsorption on a blank Au(111) surface (bottom electrode) in 125 different orientations per protein. For each protein, 3–6 adsorption structures were selected for the preparation of protein junctions (27 in total across all six proteins). This included the structure with the highest adsorption energy, structures with Cys or Met physisorbed on the bottom electrode, structures with Cys adsorbed on the bottom electrode and Met positioned at the opposite end of the protein ready for physisorption to the upper electrode once introduced, as well as a randomly chosen structure with no Cys or Met residues adsorbed on electrodes. Surface reconstruction effects due to adsorption of Cys or Met could not be taken into account due to the limitations of the force field used. After equilibration of the chosen structures, the top electrode, a slab of Au(111), was introduced and slowly lowered toward the protein until the pressure tensor in the protein region integrated to zero. Full details on the preparation of protein junctions are given in the SI.

Representative junction structures are shown in Figure 1. While the smallest proteins exhibit a globular structure (MtoD and DHC2) with the heme(s) distant from the electrodes in all orientations, the larger proteins adopt an increasingly “wire-like” shape directing their terminal heme groups toward the two electrodes. Yet, amino acids on their protein surface usually prevent direct interaction of hemes with the electrode surface. Only for one structure of the hexaheme protein GSU we find that the terminal hemes interact directly with the electrodes. The protein structures in the junctions are fairly stable in MD simulations at 300 K exhibiting an RMSD of backbone atoms between 3 and 5 Å relative to the crystal structure (with one STC configuration showing a larger RMSD), see Figures S1–S7. The RMSD values at the higher end are due to partial unfolding of the polypeptide chains that interact with the gold electrodes.

Coherent tunneling currents are calculated within the Landauer Büttiker formalism^54^

1 $$I\left(V\right)=\frac{e}{\pi \hbar }\int T\left(E\right)\left[f_{L}\left(E,V\right)-f_{R}\left(E,V\right)\right]\;dE$$

where *I* is the current, *V* the voltage, *f*_*M*_, *M* = L,R the Fermi–Dirac distribution for occupation of the electronic energy levels of the left (L) and right (R) electrodes, and *T* the transmission function calculated in the Breit-Wigner (BW) approximation^54^

2 $$T\left(E\right)=\underset{\alpha \alpha^{\prime }}{\sum }T_{\alpha \alpha^{\prime }}=\mathrm{Tr}\left[{\mathrm{\Gamma ̂}}^{\left(L\right)}\left(E\right){Ĝ}^{B†}\left(E\right){\mathrm{\Gamma ̂}}^{\left(R\right)}\left(E\right){Ĝ}^{B}\left(E\right)\right]\approx \overset{protein}{\underset{j}{\sum }}\frac{\Gamma_{j}^{\left(L\right)}\left(E\right)\Gamma_{j}^{\left(R\right)}\left(E\right)}{{\left(E-\varepsilon_{\Sigma_{j}}\right)}^{2}+\Gamma_{j}^{2}\left(E\right)}$$

where ${Ĝ}^{B}$ is the Green’s function of the bridge (protein) and ${\mathrm{\Gamma ̂}}^{\left(M\right)}$ , *M* = L,R minus twice the imaginary part of the self-energy.

In the BW approximation, the contribution of each protein orbital *j* to the current is governed by the spectral density functions $\Gamma_{j}^{\left(L\right)}\left(E\right)$ and $\Gamma_{j}^{\left(R\right)}\left(E\right)$ describing their electronic coupling with the electronic band states on the left and right electrode at energy *E*, the spectral width parameter $\Gamma_{j}\left(E\right)=\frac{1}{2}\left[\Gamma_{j}^{\left(L\right)}\left(E\right)+\Gamma_{j}^{\left(R\right)}\left(E\right)\right]$ , and the DFT+Σ corrected single-electron orbital energies of the protein, $\varepsilon_{\Sigma_{j}}$ . $\Gamma_{j}^{\left(L\right)}\left(E\right)$ , $\Gamma_{j}^{\left(R\right)}\left(E\right)$ and $\varepsilon_{\Sigma_{j}}$ are obtained from Kohn–Sham (KS) DFT calculations on the full Au-protein-Au junction using the PBE functional, followed by transformation of the KS orbitals to local orbitals localized on the protein and the left and right electrodes, respectively, using the projector operator-based diabatization method (POD).^55^ The resultant orbital energies of the protein (*ε*_*j*_) were then corrected for surface polarization effects and self-interaction error of the density functional using the DFT+Σ ^51–53^ method, giving $\varepsilon_{\Sigma_{j}}$ . The Σ correction for the occupied protein orbitals is −1.2 eV, which amounts to a down-shift of the occupied protein orbitals with respect to the Fermi level. After the shift, the highest occupied protein orbital is typically 1.2 eV below the Fermi level of the electrodes at zero voltage, *E*_F_, in agreement with photoemission spectroscopy for STC adsorbed on Au.^11^ Full details of the coherent tunnelling calculations are given in the SI.

The transmission functions for a single protein configuration in the junctions depicted in Figure 1 are shown in Figure 2a for the range −0.5 to 0.5 eV relative to *E*_F_ (see Figures S8–S13 for all protein junctions investigated). They vary smoothly because they originate from the smoothly varying tails of the Lorentzians centered at the off-resonant protein energy levels. Integration of the transmission functions over the Fermi-window according to 1 gives the coherent tunneling currents, Figure 2b. We obtain a nearly linear (Ohmic) increase in the current as a function of the applied potential up to ± 0.5 V for all junctions. Nonlinear (super-Ohmic) behavior sets in at higher voltages where one integrates over larger parts of the nonlinear Lorentzian tails. The currents tend to decrease with increasing protein size, reaching a few nA for the smaller proteins and a few 0.1 nA for the largest hexa-heme protein GSU, at 0.5 V. However, the current mainly correlates with the junction width as determined by protein size *and* orientation (see below), not by protein size only, or number of heme cofactors.

**Figure 2:**
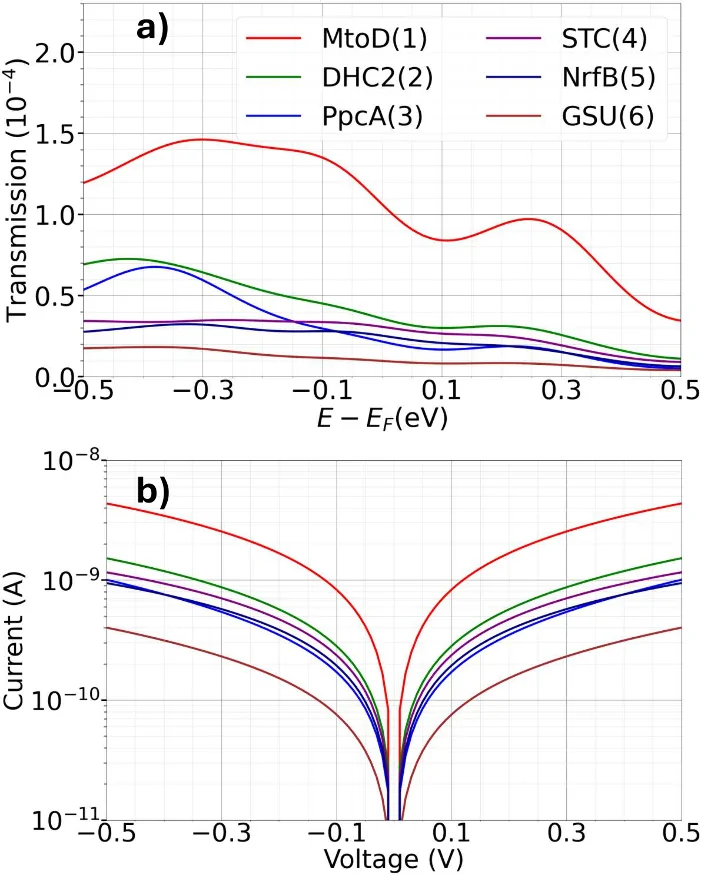
Electronic characterization of the multiheme cytochrome junctions shown in Figure 1. (a) The transmission function eq 2 and (b) the current–voltage response eq 1 are obtained from DFT+Σ calculations, see main text and SI for details. Notice the absence of sharp peaks and the low values in the transmission function since the protein energy levels are off-resonant, below −1.2 eV with respect to the Fermi level *E*_F_. Notice also the steady decrease of the transmission function and current from monoheme protein MtoD to hexa-heme protein GSU. The number of hemes present in each protein are given in parentheses in the legend.

Proteins are strongly fluctuating structures and experimental conductances are an average over a very large number of protein configurations. Given the high computational cost of DFT+Σ calculations on whole protein junctions, extensive thermal sampling of protein conductances is unfeasible. Instead, we estimated the impact of thermal protein fluctuations on conductance by monitoring the number of contacts between amino acid residues and the electrodes along MD trajectories and selecting three protein configurations per junction for the conductance calculation, ones with minimum, maximum and an average contact number. Here, the contact number *N* is the number of protein heavy atoms (C, S, O, N) within van-der-Waals distance to any of the two electrodes. The average of the three conductance values are shown in Figure 3a for each protein and orientation (27 in total). Error bars represent the difference between the smallest and the largest of the three conductance values, not the width of the conductance distribution, which would require exhaustive sampling of thermal configurations. For STC the conductance values are very similar to the ones reported previously for STC in water (typically within a factor of 2).^15^

**Figure 3:**
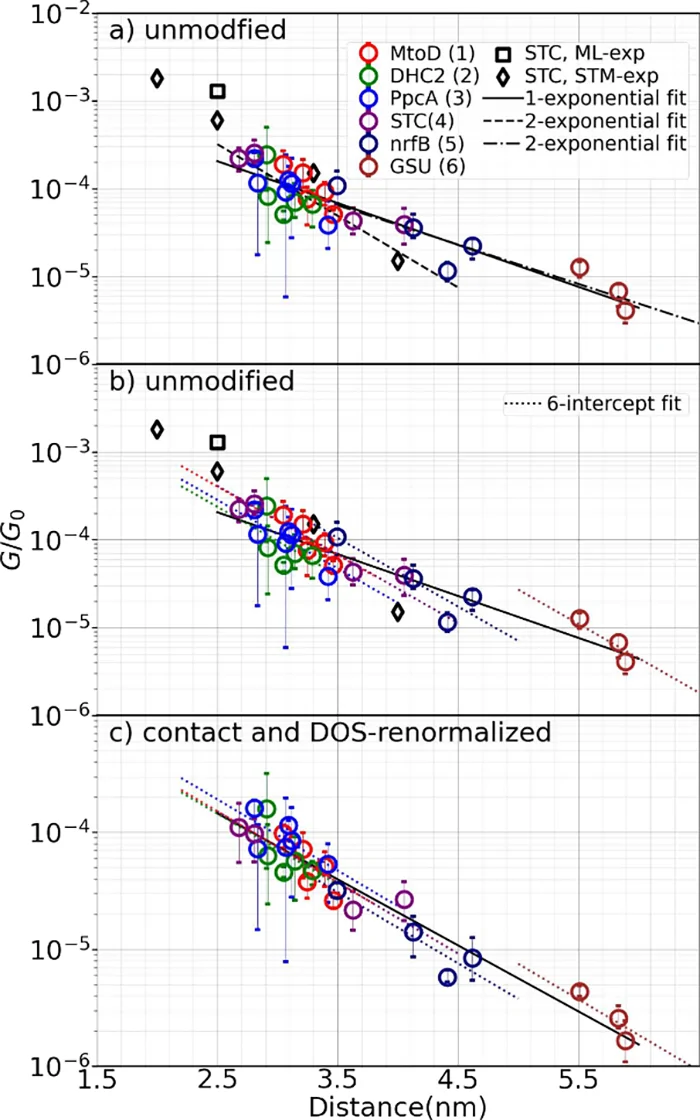
Distance decay of electronic conductance in six multiheme cytochromes. (a) Conductance for mono- to hexaheme proteins. For each of the six proteins 3–6 data points are shown corresponding to different orientations of the protein in the junction resulting in different electrode-electrode distances, i.e. junction widths. The conductance in a given protein orientation is the average over three different protein configurations with minimum, maximum and average number of protein-electrode contacts as obtained from 10 ns MD trajectory for each protein orientation. The difference between the smallest and the largest of the three conductance values is indicated by an error bar. The conductance was obtained from the slope of the respective *I*-*V* curves at *V* = 0 V. Experimentally determined conductances for STC in monolayer (ML) protein junctions^10^ and in single molecule STM junctions with fixed electrode-electrode separation^15^ are shown in black squares and diamonds, respectively. Best fits to a 1-exponential decay model (*G*/*G*_0_ ∝ exp­(−*βr*)) are shown in solid lines (β = 1.1 nm^–1^, *R* ^2^ = 0.85) and best fits to a 2-exponential decay model with crossover distance at 3.5 nm are shown in dashed and dash-dotted lines (β = 1.9 nm^–1^, *R* ^2^ = 0.54 and β = 1.0 nm^–1^, *R* ^2^ = 0.85). (b) Same data as in panel (a) with the 2-exponential distance decay fit replaced by a protein-specific 6-intercept model with a single exponential distance decay constant for all six proteins (β = 1.8 nm^–1^, *R* ^2^ = 0.91). (c) Conductance normalized by number of contacts and the density of protein electronic states, see main text for details. The normalized conductances are fit to a 1-exponential distance decay model (β = 1.3 nm^–1^, *R* ^2^ = 0.92) and the protein-specific 6-intercept model (β = 1.4 nm^–1^, *R* ^2^ = 0.94). According to AIC, the 6-intercept model in panel (b) is the statistically preferred model to describe the unmodified conductances (see Figure S14).

We investigated three different models to describe the conductance data in Figure 3a. In the first model we fit all data to a single uniform exponential decay function (1-exponential model), *G*/*G*_0_ ∝ exp­(−*βr*), giving an exponential decay constant β = 1.1 nm^–1^ (*R* ^2^ = 0.85, solid line). Visual inspection of the fit suggests that the conductance values at smaller distances <3.5 nm tend to decay faster than for larger distances. Thus, we fit the data for distances <3.5 nm and >3.5 nm separately giving an exponential decay constant β = 1.9 nm^–1^ (*R* ^2^ = 0.54, dashed line) and β = 1.0 nm^–1^ (*R* ^2^ = 0.85, dash-dotted line), respectively. The 2-exponential and 1-exponential decay models are compared using the Akaike information criterion (AIC). We find that while the 2-exponential decay model has a reduced sum of square residuals, the difference in AIC between the two models is very small and statistically insignificant, ΔAIC = AIC (2-exponential) – AIC (1-exponential) = 0.3. This remains true when the crossover distance for the 2-exponential decay fit is varied within a wide range of junction widths between 3.0 and 4.5 nm. For any crossover distance we obtain −2 ≤ ΔAIC ≤ 2, see Figure S14a (red line). This means that neither the 2-exponential nor the 1-exponential decay model describes the data better than the other. The third model we investigate is a protein-specific intercept model: the proteins share the same distance decay constant but each of the 6 proteins has its own fitted intercept. This 6-intercept model gives β = 1.8 nm^–1^ (Figure 3b dotted lines, total *R* ^2^ = 0.91). The intercepts (vertical shift) of the fit lines steadily increases with protein size suggesting that the intrinsic conductance, that is, the conductance at a given junction width, increases with increasing protein size. We find that ΔAIC = AIC­(6-intercept, β = 1.8 nm^–1^) – AIC­(1-exponential, β = 1.1 nm^–1^) = −4.9 which means that the 6-intercept model is statistically preferred over the 1-exponential model, see Figure S14b (red line).

We hypothesize that the increase in the intercept with protein size could be due to their larger footprint on the electrodes and their higher density of protein electronic states. To investigate the effect of the protein footprint, we normalize the conductance *G* of each protein junction with respect to the contact number *N*, $G\to G\frac{N_{\mathrm{DHC}2}}{N}$ , where *N*_DHC2_ is the contact number-averaged over 5 DHC2 junctions (5 different orientations, 1 configuration each with minimum contact number). We chose to normalize the contact number to the one of DHC2 because it is the smallest of the 6 proteins investigated here in terms of number of amino acids. Though the qualitative results are largely independent of the choice of reference protein. We find that the average total contact number of the different proteins, i.e., their footprint on the two electrodes, is rather similar despite their different size, varying between 17 and 25. Thus, contact number renormalization only has a very small effect on the conductance and cannot explain the protein-specific intercepts.

To investigate the effect of the density of states, we renormalize the conductance *G* by the contact number *N* and the density of protein electronic states, ρ­(*E*)­

$$G=\int dE\;G\left(E\right)\to \int dE\;G\left(E\right)\frac{N_{\mathrm{DHC}2}}{N}\frac{\rho_{\mathrm{DHC}2}\left(E\right)}{\rho \left(E\right)}$$

where *G*(*E*) is the contribution of protein electronic states with energy *E* to the total conductance *G* and ρ_DHC2_(*E*) is the density of protein electronic states averaged over all DHC2 junctions (5 different orientations and 3 different configurations per orientation, 15 in total). Thus, the conductance for each protein is normalized to the contact number and density of protein electronic states that is characteristic of a DHC2 protein junction. The renormalization leads to a significant decrease in conductance for the larger proteins relative to the smaller proteins, see Figure 3c. The renormalized conductances are now better described by a single uniform exponential decay (Figure 3c solid lines, β = 1.3 nm^–1^, *R* ^2^ = 0.92) than the original unmodified data (Figure 3a solid lines, β = 1.1 nm^–1^, *R* ^2^ = 0.85). The 6-intercept model gives β = 1.4 nm^–1^ (Figure 3c dotted lines, total *R* ^2^ = 0.94) and no longer has a statistical preference over the 1-exponential model, ΔAIC = AIC­(6-intercept, β = 1.4 nm^–1^) – AIC­(1-exponential, β = 1.3 nm^–1^) = −0.8, see Figure S14b (green line). This suggests that the varying density of protein electronic states explains (at least partially) the increasing intercepts with protein size.

Another intriguing feature of the data shown in Figure 3 is that the range of conductance values for minimum and maximum contact numbers (indicated by error bars in Figure 3a) are fairly large for the smaller proteins but steadily decrease with increasing protein size. We note that this is still the case after contact and density of state renormalization (Figure 3c). This suggests that intraprotein thermal fluctuations are a likely cause for this feature affecting the conductance of smaller proteins more strongly than the one of larger proteins. For further analysis, we break down the conductance in contributions from the different protein channels. In Figure 4, we show the distribution of the current contributions from the different protein channels for PpcA (Figure 4a) and GSU (Figure 4b) representing junctions with high and low thermal fluctuations in conductance. For PpcA, the conductance distributions were relatively narrow. In some configurations, we obtain high conductance (Figure 4a, data in green) due to strong peaks from highly delocalized protein channels (i.e., protein orbitals) that electronically connect the two electrodes very well (depicted in Figure 4c). In other configurations, these high conductance channels no longer exist due to thermal motion resulting in a strong decrease in the current (Figure 4a, data in red). By contrast, in GSU, the conductance distributions are broader and more similar to one another (Figure 4b). High conductance channels (e.g., Figure 4d) do break as a consequence of thermal motion, but they are more easily replaced by other conductance channels due to the higher density of electronic states and contact number.

**Figure 4:**
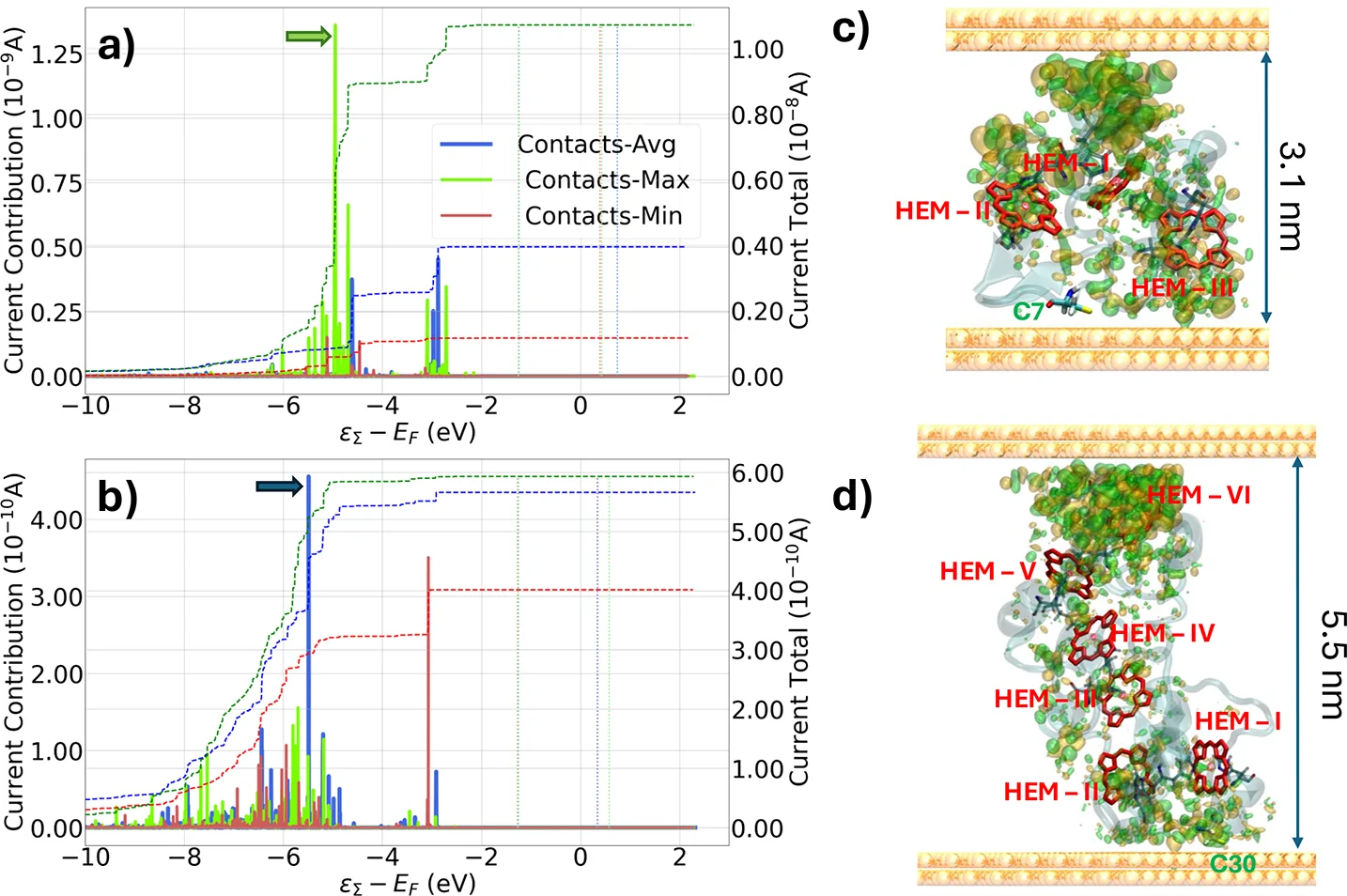
Breakdown of tunneling current in contributions from protein conductance channels. The contribution of the protein orbitals to the total current is shown as a function of the protein orbital energy *ε*_Σ_ relative to the Fermi-level *E*_F_ for a PpcA (a) and a GSU junction (b) (vertical sticks, *y*-axis to the left). The current contributions were obtained by integrating the transmission function for each orbital *j* in eq 2 over the Fermi window from −0.5 V to +0.5 V according to eq 1. The accumulated sum of the contributions is denoted total current (dashed lines, *y*-axis to the right). For each protein the breakdown is shown for three different protein configurations with minimum (red), maximum (green) and average number of contacts (blue) between protein residues and electrodes as obtained from MD simulation. For both PpcA and GSU a protein orientation was chosen where a Cys residue (PpcA:C7 and GSU:C30) is chemisorbed on the bottom electrode. Notice the larger variation in current contributions and in total current for the three configurations for PpcA than for GSU. The protein orbital with the largest current contribution is indicated by an arrow in (a) and (b), and isosurfaces of these orbitals are shown in (c) and (d), respectively. For the orbital shown in (c), *I*_*j*_ = 1.4 × 10^–9^ A, *ε*_Σ,*j*_ = −5.0 eV, Γ_L_ = 31 meV, Γ_R_ = 18 meV and for orbital shown in (d), *I*_*j*_ = 4.6 × 10^–11^ A, *ε*_Σ,*j*_ = −5.5 eV, Γ_L_ = 23 meV, Γ_R_ = 18 meV.

Finally, we investigate how different protein modifications affect the conductive behavior. We considered the same junctions as before and replaced the Fe atoms of the hemes by 2 hydrogen atoms and recalculated the conductance. The effect is very small for all proteins, a change in conductance by less than a factor of 2 compared to the unmodified protein, except for DHC2 where the conductance drop is somewhat larger (Figure 5, diamonds). The small effect of iron removal is consistent with an experimental study on horse-heart Cyt-C where both native and mutant enzymes without iron present could be investigated.^56^ Removal of all heme cofactors had a larger effect, a reduction in conductance by a factor of 2–6 (Figure 5, squares). Yet, these “apo” proteins are still fairly conductive because a significant fraction of the conduction channels do not involve heme groups. Lastly, removing all protein amino acids and keeping only the heme cofactors along with the cysteine linkages and axial histidine residues, the conductance decreases dramatically, or in some cases, vanishes alltogether (Figure 5, pentagons). This is because the hemes do not interact strongly with the electrodes and removal of all amino acids leaves large vacuum gaps prohibiting significant tunneling.

**Figure 5:**
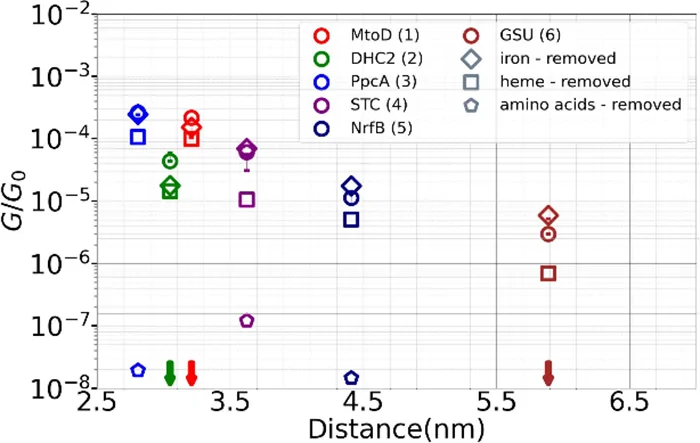
Impact of protein modifications on conductance. For each unmodified protein an orientation was selected where the contribution of Fe-hemes to the total conductance was the highest (circles). The conductance was then recalculated for these structures after (i) replacing each Fe atom by 2 H atoms (diamonds) (ii) removing all hemes along with axial histidines and termination of cysteine linkages by SH (squares) (iii) removing all protein residues except axial histidines (pentagons). In modification (iii), an arrow pointing downward denote a conductance *G*/*G*_0_ < 10^–8^.

In summary, we found that the computed conductance decay for the six multiheme cytochromes is best described by a protein-specific intercept model with a single exponential distance decay constant of 1.8 nm^–1^ and an increasing intercept with protein size. We also found that as the protein size increases the distribution of protein conductance channels widens which makes the conductance of large proteins less sensitive to random protein thermal fluctuations at the protein-electrode interface. Both of these results can be explained by the increasing density of protein electronic states with protein size. Intriguingly, Fe appears to be unimportant and heme cofactors appear to have a smaller than expected role for electronic conduction within these junctions: the proteins would still conduct without hemes being present if they were structurally stable without hemes bound. Hence, our results imply that recent Au-multiheme protein-Au junction measurements in vacuum^10^ and in water^15^ mainly probe the electronic states of the polypeptide chain. In other words, they do not probe the natural physical process that occurs in the bacterial cell, heme-to-heme electron hopping.^30,31^ This also clarifies why hopping models cannot explain the high and near temperature-independent currents obtained from junction measurements.^11,12,24^ Although we have only studied six MHC proteins here, we believe that the above findings are applicable to other MHCs of comparable size.

It will be of interest to see whether future experimental junction measurements on native and de novo designed multiheme cytochromes will find further evidence for a nonuniform conductance decay with increasing protein size, as predicted in the present work. It will also be of interest to see whether a change in the conduction mechanism in multiheme cytochrome junctions can be induced from off-resonant coherent tunneling to heme-to-heme hopping by shifting the heme redox levels closer to the electrode Fermi levels through application of a gate voltage.

The off-resonant coherent tunneling mechanism invoked here to explain the conductance of small to medium-sized MHCs has well-known limitations. In particular, it neglects the coupling between the electronic and nuclear degrees of freedom and it cannot explain the unusually high conductances recently reported for bacterial filaments made of the polymerized MHC OmcZ.^57^ These filaments span hundreds of nanometers and were reported to support nA currents at 0.1 V. For comparison, the largest MHC studied here, GSU, is about 5.5 nm large and is predicted to support currents of about 0.1 nA at 0.1 eV. Heme-to-heme hopping cannot explain the high conductances of these fibers either because pump–probe transient absorption spectroscopy and computation have shown that heme-to-heme hopping times in MHCs are no faster than nanoseconds, even for tightly stacked heme pairs at van-der-Waals distance.^31^ Hence, if the reported measurements for OmcZ-based bacterial filaments stand the test of time, other mechanism will need to be invoked to explain their high conductances, e.g. the transient quantum delocalization mechanism, that has been put forward to explain charge transport in high-mobility organic semiconductors.^58^

## Supplementary Material



## Notes

Full computational details on the preparation of protein junctions, calculation of *I*-*V* curves and conductances, figures showing all junctions, their RMSD wrt crystal structure, transmission functions, *I*-*V* curves, breakdown of current contributions, AIC analysis, and density of protein electronic states, and tables summarizing conductances and electronic properties of the junctions (PDF)
