# Supplementary material for: Nonuniform Distance Decay of Conductance in a Series of Multiheme Cytochrome Bioelectronic Junctions
Source: J Phys Chem Lett. 2026 Jul 31;17(32):9145–52. doi: 10.1021/acs.jpclett.6c01449 (PMC13488441; doi:10.1021/acs.jpclett.6c01449)
Supplement: Supplementary file 1 [file jz6c01449_si_001.pdf]

Supporting Information: Non-uniform Distance Decay of Conductance in a Series of Multi-heme Cytochrome Bioelectronic Junctions

A. M. Pethő,[†] Zdenek Futera,[‡] and Jochen Blumberger[*,†]

†*Department of Physics and Astronomy and Thomas Young Centre, University College London, London, WC1E 6BT, United Kingdom*

‡*Faculty of Science, University of South Bohemia, 370 05 Ceske Budejovice, Czech Republic*

E-mail: j.blumberger@ucl.ac.uk

Full Computational Details

Force field. MD simulations for proteins in vacuum and for protein junctions were carried out with the golp-charmm-27 forcefield.[1] The redox state of the protein under vacuum conditions is not known experimentally. We have chosen to model all heme-cofactors in the reduced state for computational reasons. Fe(II)-heme is closed shell singlet and more reliably modelled by the PBE functional than the Fe(III) low spin doublet state. The forcefield parameters for Fe(II)-heme c were taken from Ref.[2] and the ones for chemisorbed cysteine from Ref.[3] The golp-charmm-27 forcefield uses dummy atoms to model the induced electronic dipoles of the Au atoms of the Au(111) surface and their interaction with the adsorbed amino acids.[3] When simulating a Au(111) slab with this method, the Au atoms were frozen, while the dummy atoms were propagated at a constant temperature of 300 K during all equilibration and production runs. All MD simulations were carried out using the Gromacs MD software.[4,5]

Protein MD simulations in vacuum. The starting point for preparation of the protein junctions was the protein crystal structures deposited in the pdb database: MtoD:4xxl,[6] DHC2:2czs,[7] PpcA:2mz9,[8] nrfB:2ozy[9] and GSU:[10]3ouq. For STC we used the junctions from Ref.[2] that are based on the structure from pdb id 1m1q with mutation S89C. All proteins have one or more Fe heme-c cofactor covalently linked to the polypeptide chain by a CXYCH motif. With the exception of the GSU (where 2 of 6 hemes are Met-His coordinated), all heme cofactors are ligated by two axial His residues. Crystal waters were removed and protonation states modified in accord with vacuum conditions. That is, all ionizable non-terminal residues were treated in the neutral protonation state except when they formed salt bridges. The N-terminal amino acids were capped with an acetyl (ACE) group and the C-terminal amino acid with an amine group (NHE). All hemes were treated in the reduced state.

For each system the following equilibration procedure in vacuum was carried out: we placed the protein into a cubic box with side length 15 nm applying 3D periodic boundary

conditions. In the first 5 ps the MD time step was set to $\Delta t = 0.25$ fs and a target temperature was set to $T = 150$ K using velocity rescaling. The positions of the protein heavy atoms were frozen and the hydrogens were allowed to relax. For the next simulation, we increased the temperature to 300 K and the timestep to $\Delta t = 1$ fs and run the system using a Berendsen thermostat with the same position constraints for a further 50 ps. The next three simulations of length 50 ps used the same parameters, but instead of a constraint, the protein heavy atoms were restrained with a harmonic potential with force constant of 1000, 500 and 50 kJ/mol/nm^2, respectively. This was followed by a simulation without the restraints for 50 ps. In the penultimate step we have switched the thermostat to Nose-Hoover and ran a further 100 ps of dynamics. Lastly, we ran a 10 ns production run with the Nose-Hoover thermostat and tracked the RMSD of the system during the simulation. Then the following point mutations were carried out for each system to introduce favourable S-containing binding sites in the proteins: MtoD: L45M and S81C, DHC2: T37C, PpcA: K7C,[11] NrfB: A26C, GSU: A30C. These protein mutation sites would be chosen in a typical workflow for preparation of experimental junctions. The mutated systems were re-equilibrated repeating the procedure described above.

Generation of protein junctions. In the next step a $34 \times 38 \times 6$ Au(111) slab was introduced at the bottom of the simulation cell ("bottom electrode") for each protein using 3D periodic boundary conditions. To simulate the adsorption of the protein onto the surface, we generated 125 initial protein orientations and placed them 10 Å above the Au surface. The initial protein orientations were obtained by first orienting the protein such that the Cys residue points downwards to the Au slab followed by rotation of the protein in increments of ±22.5 degrees about the axis perpendicular to the Au surface and the two axes parallel to the surface. Rotations about all three axes was from 0 to ±45 degrees.

We applied the following simulation protocol consisting of 5 steps for each of the systems. First, the structure was relaxed during a 0.5 ps MD run with a timestep of 0.1 fs and at 150 K using velocity rescaling. This was followed by a 5 ps run with a time step of 0.5 fs

and at 300 K using a Berendsen thermostat, a 10 ps run with a Nose-Hoover thermostat at 300 K and a 10 ns run with $\Delta t = 1$ fs timestep at 300 K using a Nose-Hoover thermostat. Lastly, we ran a short 100 ps simulation to extract the energy of the Au-protein complex for calculation of the protein adsorption energy.

The 125 adsorption structures were arranged in three groups. Group 1 had a Cys, group 2 a Met and group 3 neither a Cys or a Met physisorbed to the bottom electrode (noting that in several configurations the Cys initially pointing towards the bottom electrode no longer does so at the end of the MD equilibration). For MtoD, DHC2 and PpcA we chose two structures from each of group 1 and 2, one that had the highest adsorption energy in their group (structures **1**, **2**) and ones where the axis between the physisorbed Cys (Met) and a Met (Cys) at the opposite end of the protein is close to the surface normal (structures **3** (**4**)). One structure was chosen from group 3, the one with the highest adsorption energy in that group (structure **5**). In case of the PpcA system a further configuration from group 3 was chosen where the heme chain ran from the bottom to the top electrode (structure **6**). For NrfB, 4 structures were selected using the same criteria used to select structures **1**, **3**, **4** and **6** for the smaller proteins above. For GSU, 3 structures were selected using the same criteria used to select structures **3**, **4** and **6** for the smaller proteins above. Configurations corresponding to structures **2**, **5** were not stable for NrfB and GSU. For STC we chose the 4 structures from previous work denoted L1, L2, S1 and S2.[2] In total, we selected 27 adsorption structures across the 6 proteins.

To prepare junction structures we decreased the box size along the surface normal such that the upper part of the protein adsorbs on the bottom face of the periodic replica of the Au(111) slab along the surface normal (while the lower part of the protein remains adsorbed on the top face of the slab in the central cell). Initially, the box size was adjusted such that the distance between the atom closest to the box edge and the bottom face of the periodically replicated slab was 2 Å. Then the same 5-step equilibration protocol was applied as described above, though in the 5th step the 100 ps simulation was replaced by

a 5 ns simulation as required to converge the stress tensor. Then the box length along the surface normal was decreased in steps of 0.25 Å followed by 5-steps of re-equilbration until the stress tensor was zero. The distance along the surface normal at this point is referred to as the "junction width" in the main text.

Using the final junction width, each of the junctions underwent a further 10 ns of dynamics during which the number of contacts between the protein amino acids and the electrodes were tracked. The contact number N of a given protein configuration in the junction is the number of heavy atoms (C, S, O, N) within van-der-Waals distance to any of the two electrodes. We used the contact number to select three protein configurations from the 10 ns MD simulation for calculation of the current-voltage response: one with an average (denoted "avg"), one with the minimum ("min") and one with the maximum ("max") contact number. Since the protein was adsorbed first on the bottom electrode each of these configurations had a significantly larger contact number on the bottom electrode along the entire MD trajectory. The 10 ns MD runs and the selection of thermal configurations was also done for the S1, S2, L1 and L2 junctions of STC starting from the structures investigated in Ref.[2] Thus, we selected a total of $3 \times 27 = 81$ junction structures across the 6 proteins for calculation of the current-voltage characteristics.

DFT calculations of current-voltage characteristic. For DFT calculation of the current-voltage characteristic we took the structures generated from MD simulation as described above and applied the following modifications to the simulation cell. (i) the size of the Au(111) slab was reduced to $17 \times 20 \times 2$. (ii) a second explicit Au(111) slab of the same reduced dimension was introduced as a top electrode and placed at the atomic positions of the periodic image of the Au slab that interacted with the top end of the protein in the above classical MD simulation. (iii) vacuum padding of 1 nm above the upper and below the lower Au slabs was applied. In DFT calculations 3D periodic boundary conditions are still applied for this modified cell but due to the vacuum padding the interactions between periodic images along the surface normal are now vanishingly small.

The current and the transmission function are calculated as described in the main text Eqs. (1)-(2) in the main text. The protein orbital energies ϵ_{Σ_j} and spectral density functions $\Gamma_j^{(L)}$ and $\Gamma_j^{(R)}$ are calculated using the projection operator-based diabatization (POD) method implemented in the CP2K package.[12] The POD method constructs the electronic states localized on the protein and electrodes from the Kohn-Sham (KS) states

$$\hat{H} |\psi_i\rangle = \epsilon_i |\psi_i\rangle \tag{S1}$$

where the latter are obtained by standard KS-SCF calculations. The KS Hamiltonian is transformed to an orthonormal atomic orbital basis ($\{\phi_i\}$) using Lowdin orthogonalization Eq.S2

$$\tilde{H} = S^{-1/2} \cdot H \cdot S^{-1/2} \tag{S2}$$

and the orthogonal basis functions are rearranged to the following block structure

$$\tilde{H} = \begin{bmatrix} \tilde{H}_{\mathrm{LL}} & \tilde{H}_{\mathrm{LP}} & \tilde{H}_{\mathrm{LR}} \\ \tilde{H}_{\mathrm{RL}} & \tilde{H}_{\mathrm{PP}} & \tilde{H}_{\mathrm{PR}} \\ \tilde{H}_{\mathrm{RL}} & \tilde{H}_{\mathrm{RP}} & \tilde{H}_{\mathrm{RR}} \end{bmatrix}, \tag{S3}$$

where labels L, R and P stand for the left electrode, right electrode and the protein, respectively. The desired localized states are obtained by diagonalization of the diagonal blocks, $\bar{H}_{\alpha\alpha}$, $\alpha \in \{$L,R,P$\}$, while the off-diagonal blocks are transformed correspondingly:

$$\bar{H}_{\alpha\alpha} = U_\alpha^\dagger \cdot \tilde{H}_{\alpha\alpha} \cdot U_\alpha \quad \text{and} \quad \bar{H}_{\alpha\beta} = U_\alpha^\dagger \cdot \tilde{H}_{\alpha\beta} \cdot U_\beta, \quad \alpha, \beta = L, R, P \tag{S4}$$

This yields the block-diagonal KS Hamiltonian matrix in the localized basis, Eq.S5.

$$\bar{H} = \begin{bmatrix} \begin{matrix} \epsilon_{\mathrm{L},1} & \cdots & 0 \\ \vdots & \ddots & \vdots \\ 0 & \cdots & \epsilon_{\mathrm{L},N_{\mathrm{L}}} \end{matrix} & \bar{H}_{\mathrm{LP}} & \bar{H}_{\mathrm{LR}} \\ \bar{H}_{\mathrm{PL}} & \begin{matrix} \epsilon_{\mathrm{P},1} & \cdots & 0 \\ \vdots & \ddots & \vdots \\ 0 & \cdots & \epsilon_{\mathrm{P},N_{\mathrm{P}}} \end{matrix} & \bar{H}_{\mathrm{PR}} \\ \bar{H}_{\mathrm{RL}} & \bar{H}_{\mathrm{RP}} & \begin{matrix} \epsilon_{\mathrm{R},1} & \cdots & 0 \\ \vdots & \ddots & \vdots \\ 0 & \cdots & \epsilon_{\mathrm{R},N_{\mathrm{R}}} \end{matrix} \end{bmatrix} \tag{S5}$$

The off-diagonal elements of the Hamiltonian are used to obtain the spectral density functions,

$$\Gamma_j^{(M)}(E) = 2\pi \left[\left| [\bar{H}_{MP}]_{mj} \right|^2 \rho_M(\epsilon_{M,m}) \right]_{\epsilon_{M,m}=E} \quad M = \mathrm{L, R} \tag{S6}$$

where ρ_M is the density of the metallic states of electrode M.

The KS Hamiltonian of the protein junctions were calculated with the PBE[13] functional along with GTH-pseudo-potentials[14] using the CP2K programme package.[15] The basis set used was DZVP-MOLOPT-SR-GTH.[16] The protein orbital energies $\epsilon_{P,j}$ obtained from generalized-gradient approximation (GGA), such as PBE, suffer from incorrect alignment of the protein states with respect to the gold states due to the self-interaction error and missing electronic polarization of metallic surface states, respectively. These were corrected by applying the GGA+Σ scheme[17–19] as in our previous work.[2,20]

The Σ correction consists of two parts, one correcting for the self-interaction error of the functional, $\Sigma^0_{P,j}$, and one for the missing electronic polarization of the electrodes, $\Sigma^{pol}_{P,j}$,

$$\Sigma_{P,j} = \Sigma^0_{P,j} + \Sigma^{pol}_{P,j}. \tag{S7}$$

Although the corrections can be in principle evaluated for each individual electronic state, in practice $\Sigma_{P,j}$ values are determined for the HOMO and LUMO levels and used for all occupied and unoccupied levels, respectively,

$$\varepsilon_{\Sigma_j} = \varepsilon_{P,j} + \Sigma_{P,\mathrm{HOMO}} \quad \text{for} \quad j \quad \text{occupied} \tag{S8}$$

$$\varepsilon_{\Sigma_j} = \varepsilon_{P,j} + \Sigma_{P,\mathrm{LUMO}} \quad \text{for} \quad j \quad \text{unoccupied.} \tag{S9}$$

For the polarization corrections, we used the values computed in Ref.,[20] $\Sigma^{pol}_{\mathrm{P,HOMO}} = 0.031\,\mathrm{eV}$ and $\Sigma^{pol}_{P,\mathrm{LUMO}} = -0.113\,\mathrm{eV}$. The self-interaction correction was re-calculated in this work by replacing a fraction of PBE exchange by (global) exact exchange in the PBE functional and optimally tuning the fraction of exact change so as to obey Janak's theorem for the ionization potential IP, IP $= -\epsilon_{\mathrm{HOMO}}$. The optimal tuning was carried out on simplified models of the Fe-heme cofactors of STC. They were extracted from the crystal structure of STC, the heme ring substituents were replaced by H (i.e., changed to porphine rings) and the axial histidines were replaced by imidazoles. The IP calculations where carried out for the Fe(II)-heme cofactor models using PBE pseudopotentials and the same basis set used for the calculation on the full junctions. The results are summarized in Table 1. We obtain values $\Sigma^{0}_{P,\mathrm{HOMO}} = -1.225\,\mathrm{eV}$ and $\Sigma^{0}_{P,\mathrm{LUMO}} = 0.397\,\mathrm{eV}$, which are averages over all 4 heme groups and an optimally tuned fraction of exact exchange of 19%. According to Eqs. S7-S9 we applied a total shift for all occupied levels $\Sigma_{P,\mathrm{HOMO}} = -1.194\,\mathrm{eV}$ and for all unoccupied levels $\Sigma_{P,\mathrm{LUMO}} = +0.284\,\mathrm{eV}$. This amounts to a downshift of occupied protein orbitals such that the protein HOMO of STC (comprised of Fe d(t_{2g})-heme orbitals) is at $-1.2\,\mathrm{eV}$ below the Fermi-level of the electrode, E_{F} (at zero voltage), and the protein LUMO of STC (comprised of Fe d(e_g)-heme orbitals) at 1.1 eV above E_{F}. Similar positions for protein HOMO and LUMO levels are obtained for all other proteins. For the calculation of the transmission function (Eq. 2 main text) we included all occupied protein levels and all unoccupied protein levels up to an energy $\varepsilon_{\Sigma_j} = +2.384\,\mathrm{eV}$ above E_{F}. Higher lying unoccupied states tend to be highly

delocalized, which is most likely an artifact of the DFT calculations. Hence, we have not included these higher-lying unoccupied states in the current calculation. Nonetheless, it was found that unoccupied states up to +3.5 eV above E_F contribute less than 0.01% to the total current.

$\Sigma_{P,\mathrm{HOMO}}$ obtained here is similar to the one obtained previously by optimal tuning of the range separation parameter in a range-separated hybrid functional based on PBE at a fixed fraction of 20% exact exchange ($\Sigma_{P,\mathrm{HOMO}} = -1.245\,\mathrm{eV}$).[20] These previous calculations were carried out on similar cofactor models but the tuning was carried out to satisfy Janak's theorem for both IP and EA of the oxidized Fe(III)-heme cofactor. The correction $\Sigma_{P,\mathrm{LUMO}}$ obtained here is more than 1 eV smaller than the correction obtained previously from the optimally tuned-range separated functional ($\Sigma_{P,\mathrm{LUMO}} = 1.412\,\mathrm{eV}$).[20] The reason for this is unclear. We prefer to use the value determined in this work because it gives a more realistic description of the HOMO-LUMO gap of the heme cofactor models, arising from the splitting between the Fe d(t_{2g}) and Fe d(e_g) states, 2.55 eV.

Table S1: Optimal tuning of fraction of Hartree-Fock exchange (HFX) for the PBE functional for Fe(II)-heme cofactor models. $E_g^{\mathbf{PBE}}$ and $E_g^{\mathbf{OT\text{-}PBE}}$ is the HOMO-LUMO energy gap for PBE and optimally tuned PBE (OT-PBE) with fraction of HFX given in column 2. $\Sigma^0_{P,\mathbf{HOMO}}$ and $\Sigma^0_{P,\mathbf{LUMO}}$ are the self-interaction error corrections used in Eq. S7.

Cofactor	%HFX	E_g^{PBE} (eV)	$E_g^{\text{OT-PBE}}$ (eV)	$\Sigma^0_{P,\text{HOMO}}$ (eV)	$\Sigma^0_{P,\text{LUMO}}$ (eV)
Heme I	18	0.88	2.60	-1.272	0.451
Heme II	18	1.14	2.64	-1.152	0.354
Heme III	18	0.93	2.43	-1.221	0.273
Heme IV	19	0.75	2.52	-1.255	0.511
Average	N/A	0.93	2.55	-1.225	0.397

Table S2: **Electronic properties of MtoD and DHC2 junctions.**

protein	structure	$G/G_0(10^{-5})^a$	G_j/G^b	$(\varepsilon_{\Sigma_j} - E_\mathrm{F})^c$	$\Gamma_j^{(\mathrm{L})\ d}$	$\Gamma_j^{(\mathrm{R})\ d}$
MtoD	**1**	27	0.077	-4.813	37	17
		11	0.065	-4.204	39	22
		20	0.089	-4.066	43	16
	**2**	5.7	0.127	-5.009	33	17
		4.1	0.054	-4.592	34	11
		5.5	0.094	-4.783	10	21
	**3**	10	0.059	-4.784	11	50
		13	0.132	-4.378	28	26
		22	0.254	-4.666	18	62
	**4**	12	0.249	-4.501	14	52
		9.4	0.342	-2.334	15	21
		6.5	0.125	-5.248	22	19
	**5**	10	0.052	-5.719	35	13
		3.9	0.026	-3.299	5.6	29
		8.7	0.147	-4.529	17	21
DHC2	**1**	9.5	0.158	-4.581	13	58
		3.6	0.107	-4.977	31	29
		6.8	0.041	-4.122	31	11
	**2**	8.7	0.147	-4.529	17	21
		2.4	0.043	-3.453	35	6.7
		14	0.195	-4.623	12	38
	**3**	5.5	0.307	-4.619	49	23
		4.3	0.165	-5.077	15	18
		5.5	0.381	-5.045	33	32
	**4**	4.7	0.079	-5.209	19	27
		4.9	0.114	-5.314	15	23.
		11	0.142	-1.959	7.4	57
	**5**	9.6	0.022	-2.748	3.4	20
		14	0.128	-4.436	21	28
		50	0.165	-2.337	32	13

[a] Conductance in units of $10^{-5}G_0$. with: (I) maximum, (II) minimum and (II) average number of contacts.

[b] Contribution of protein orbital with highest conductance (G_j) to the total (G).

[c] Energy of protein orbital with highest conductance relative to the Fermi level.

[d] Electronic coupling of protein orbital with highest conductance to the left (L) or right (R) electrode, in meV.

Table S3: Electronic properties of PpcA and STC junctions.

protein	structure	$G/G_0(10^{-5})$	G_j/G	$\varepsilon_{\Sigma_j} - E_\mathrm{F}$	$\Gamma_j^{(\mathrm{L})\ d}$	$\Gamma_j^{(\mathrm{R})\ d}$
PpcA	**1**	22	0.127	-4.951	31	18
		2.7	0.130	-5.115	18	25
		9.4	0.115	-2.874	25	18
	**2**	18	0.081	-9.299	31	35
		7.8	0.089	-4.641	25	13
		11	0.137	-4.001	19	26
	**3**	2.9	0.051	-4.788	39	13
		0.59	0.028	-2.890	3.9	15
		24	0.377	-4.456	9.1	73
	**4**	20	0.120	-4.769	19	40
		20	0.095	-2.512	8.1	27
		26	0.107	-2.452	26	8.6
	**5**	7.3	0.068	-5.425	18	20
		2.2	0.145	-4.846	22	31
		2.1	0.052	-4.303	6.0	58
	**6**	20	0.142	-4.117	26	21
		1.8	0.035	-5.247	15	29
		13	0.085	-5.496	11	43
STC	**1**	16	0.042	-13.215	45	24
		29	0.084	-4.749	24	44
		22	0.059	-4.914	30	16
	**2**	19	0.030	-5.595	30	19
		22	0.081	-3.491	17	12
		36	0.111	-5.216	62	11
	**3**	3.5	0.169	-4.768	30	12
		2.3	0.156	-4.371	34	16
		6.0	0.180	-4.985	44	8.3
	**4**	3.8	0.024	-3.967	19	9.0
		3.1	0.051	-5.941	29	16
		6.1	0.314	-2.946	26	10

Table S4: Electronic properties of NrfB and GSU junctions.

protein	structure	$G/G_0(10^{-5})$	G_j/G	$\varepsilon_{\Sigma_j} - E_F$	$\Gamma_j^{(L)\ d}$	$\Gamma_j^{(R)\ d}$
NrfB	**1**	2.7	0.068	-4.830	14	30
		2.5	0.101	-4.986	17	22
		1.6	0.043	-4.804	4.1	33
	**2**	16	0.232	-3.530	20	17
		6.7	0.068	-2.871	2.1	30
		10	0.079	-4.228	44	9.1
	**3**	2.8	0.146	-4.957	27	12
		2.9	0.140	-5.577	21	14
		5.1	0.060	-5.580	12	18
	**4**	1.5	0.077	-3.265	21	4.0
		0.89	0.072	-5.106	27	5.1
		1.1	0.162	-5.243	8.6	36
GSU	**1**	1.5	0.026	-5.704	9.5	29
		0.98	0.087	-3.071	25	3.7
		1.4	0.081	-5.489	23	18
	**2**	0.50	0.041	-9.334	8.3	22
		0.44	0.078	-5.675	11	12
		0.30	0.018	-4.824	22	3.8
	**3**	0.77	0.068	-4.622	41	5.5
		0.45	0.089	-9.854	30	6.1
		0.82	0.102	-5.337	48	5.6

Table S5: Electronic properties modified protein junctions.

protein	structure	$G/G_0 (10^{-5})$			
		full	Fe → 2H	hemes removed	amino acids removed
MtoD	**3**	22	15	9.8	0.000
DHC2	**3**	4.3	1.7	1.4	0.000
PpcA	**4**	26	24	11	0.002
STC	**4**	6.1	6.9	1.0	0.012
NrfB	**4**	1.1	1.7	0.50	0.001
GSU	**2**	0.30	0.59	0.07	0.000

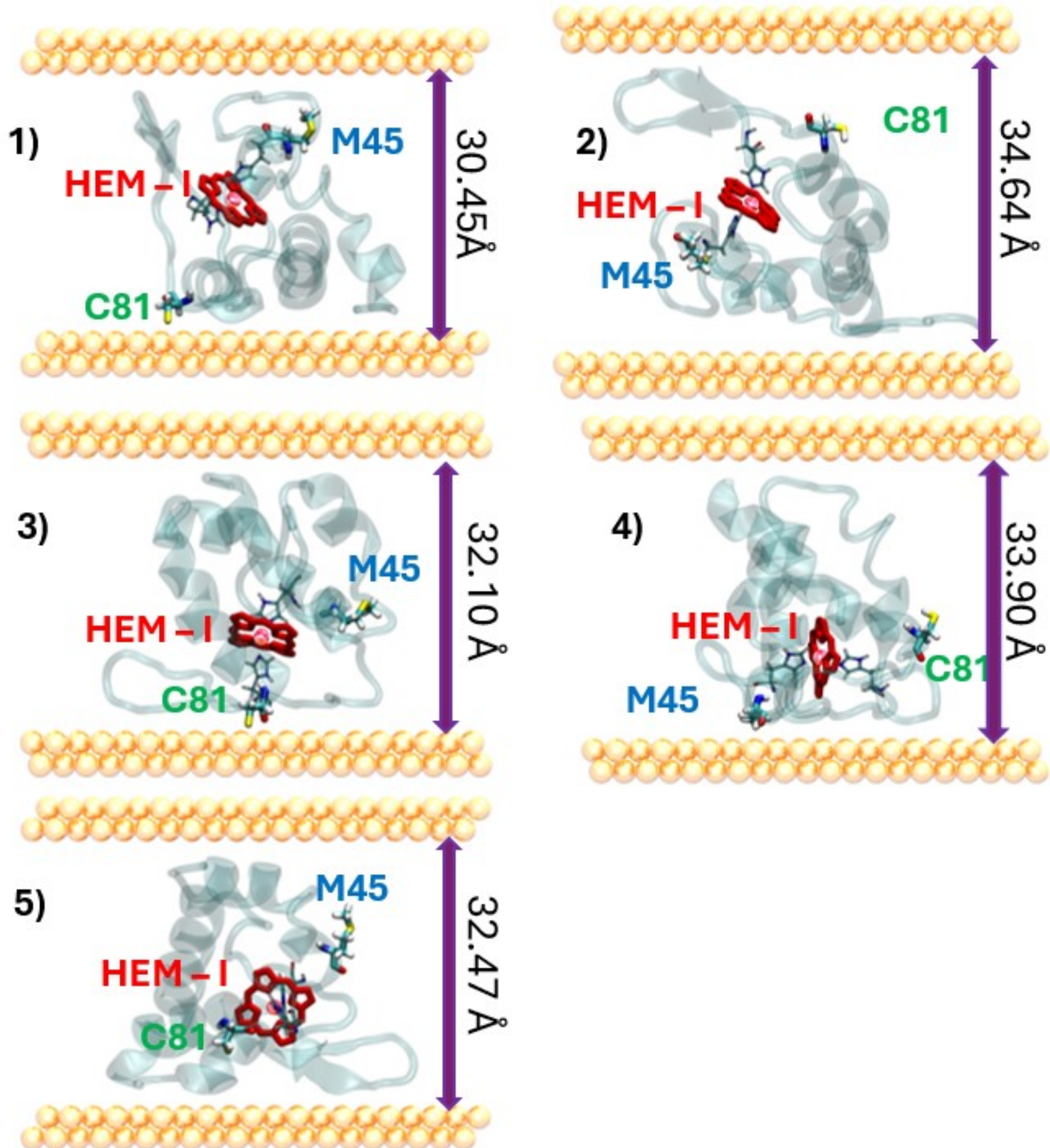

Figure S1: MtoD junction structures **1-5**.

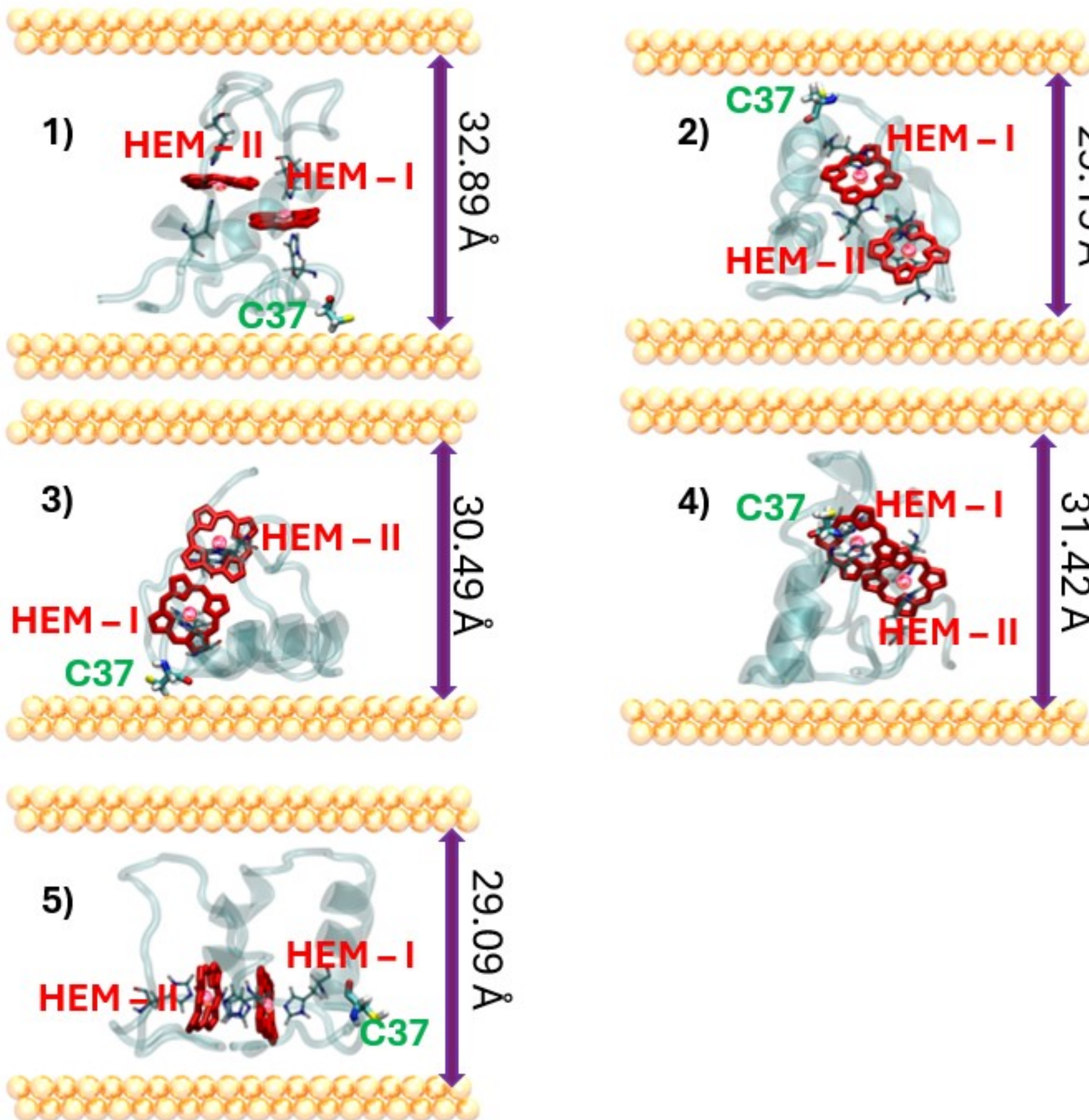

Figure S2: DHC2 junction structures **1-5**.

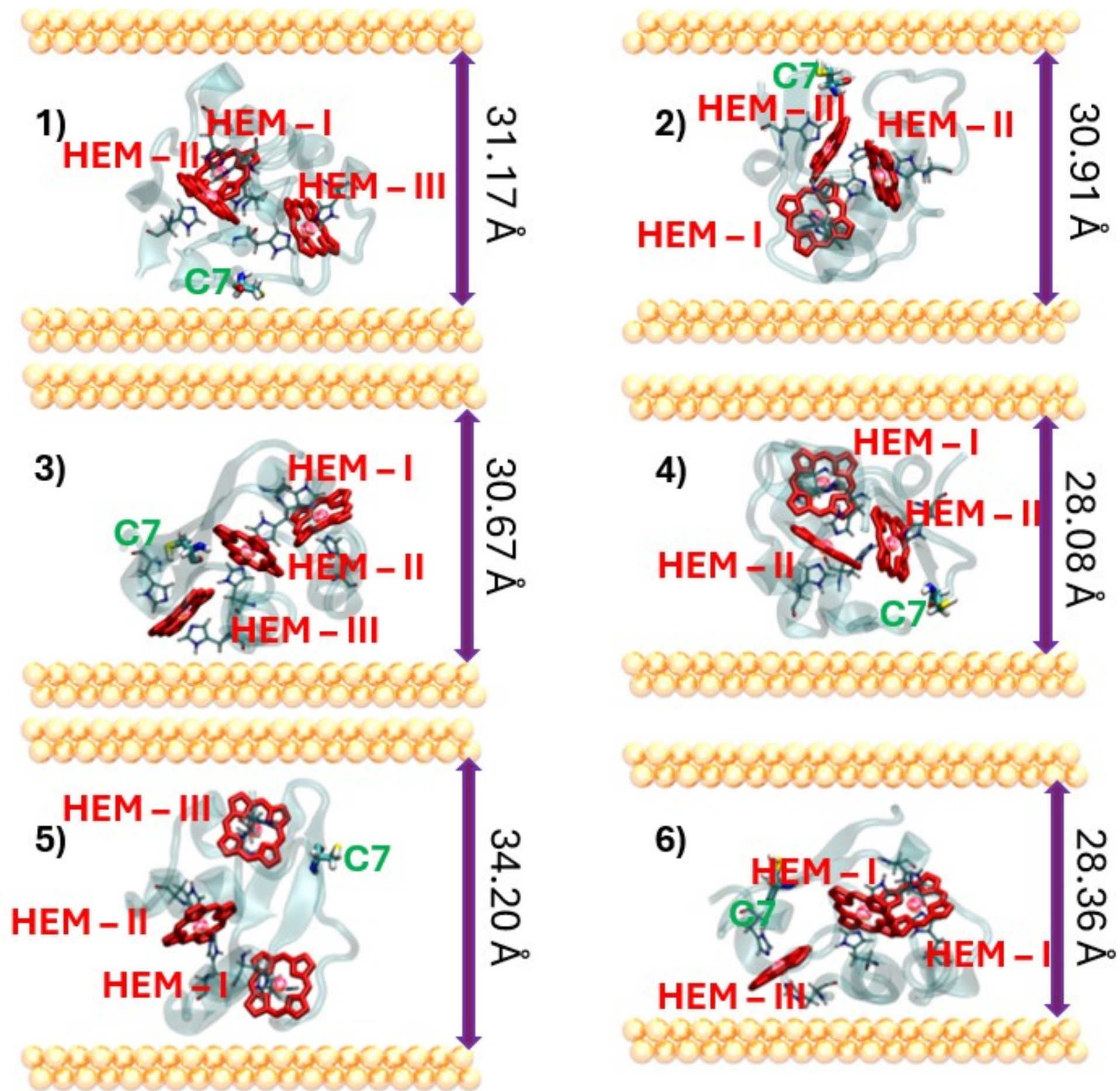

Figure S3: PpcA junction structures **1-6**.

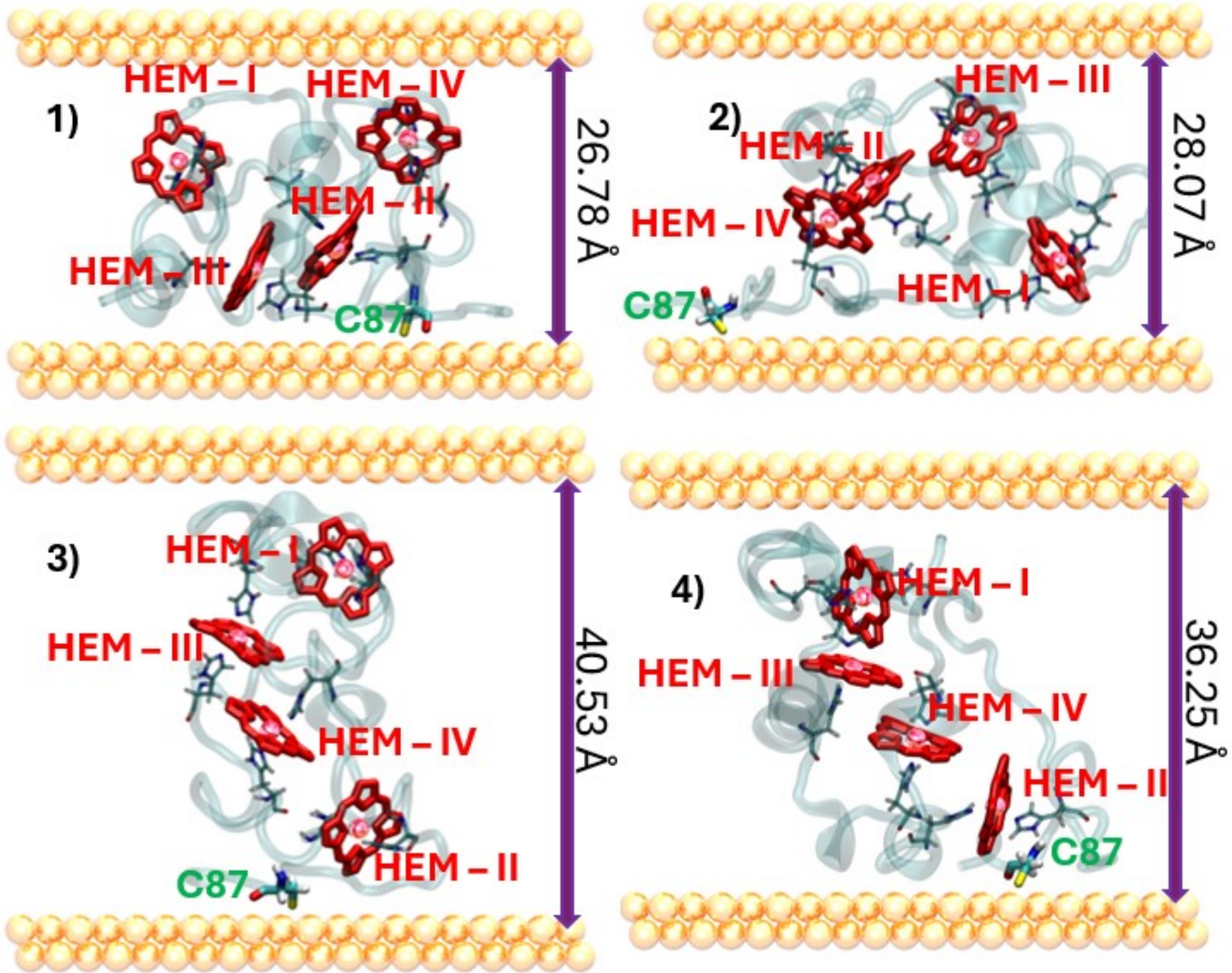

Figure S4: STC junction structures **1-4**.

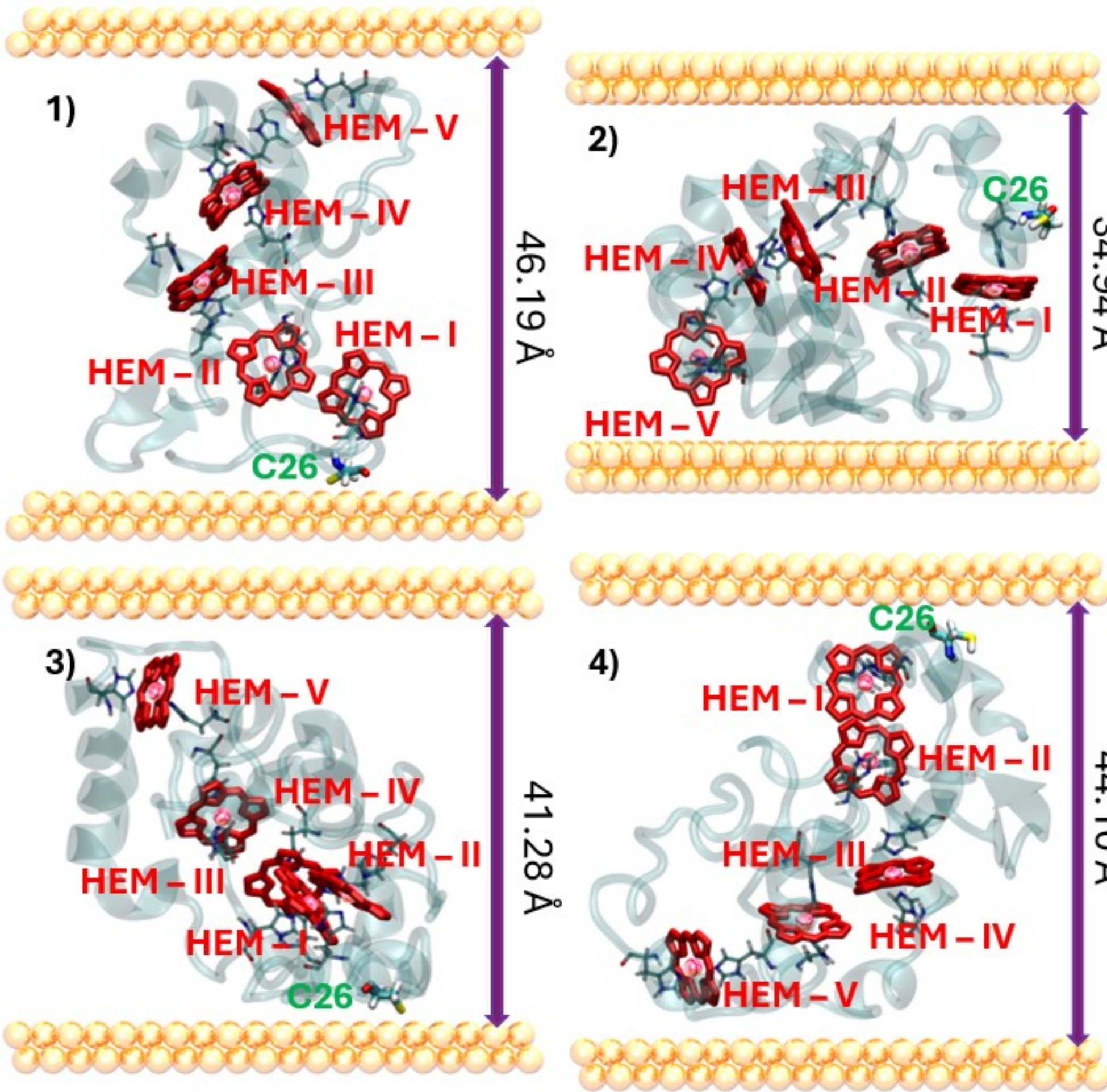

Figure S5: NrfB junction structures **1-4**.

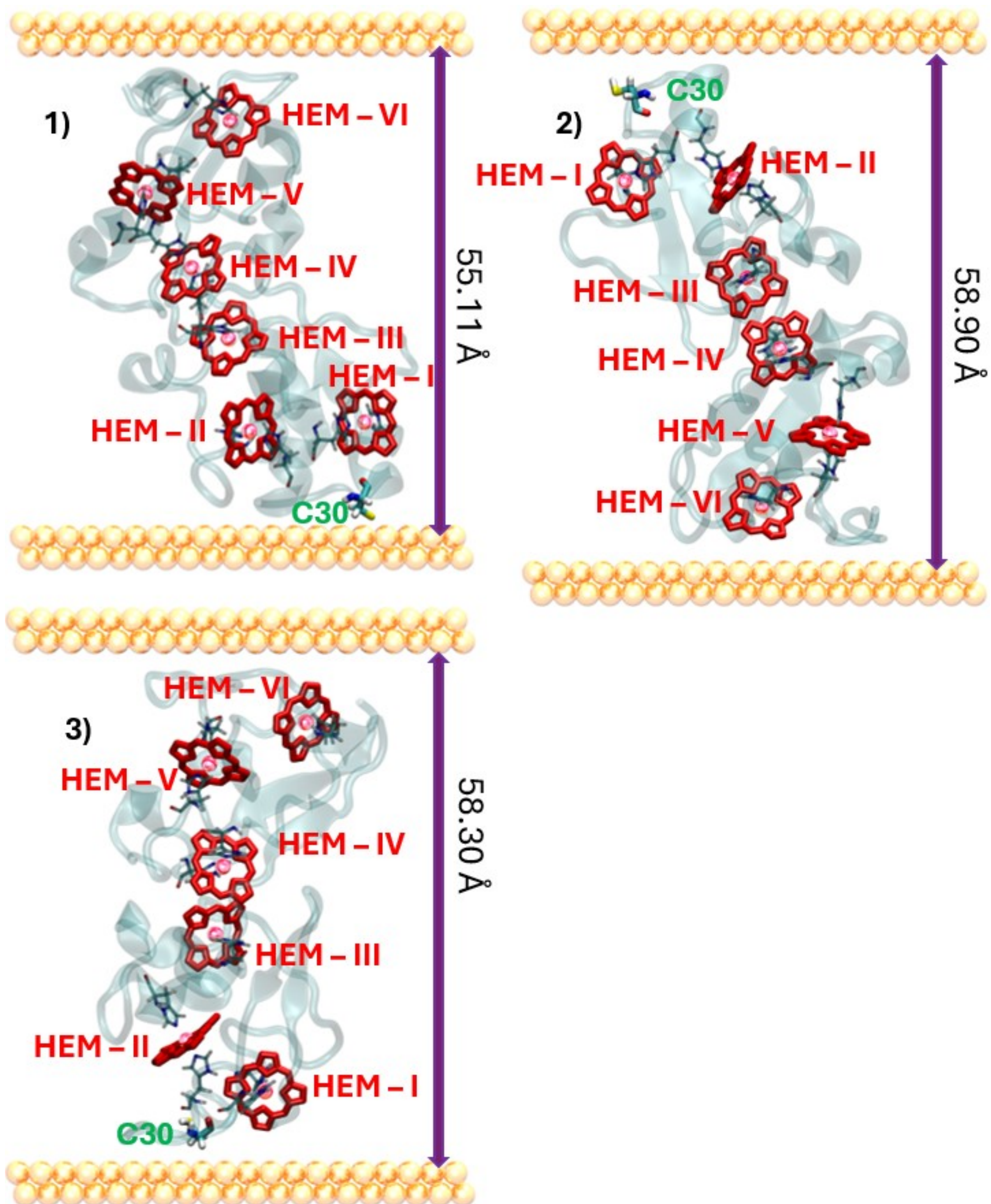

Figure S6: GSU junction structures **1-3**.

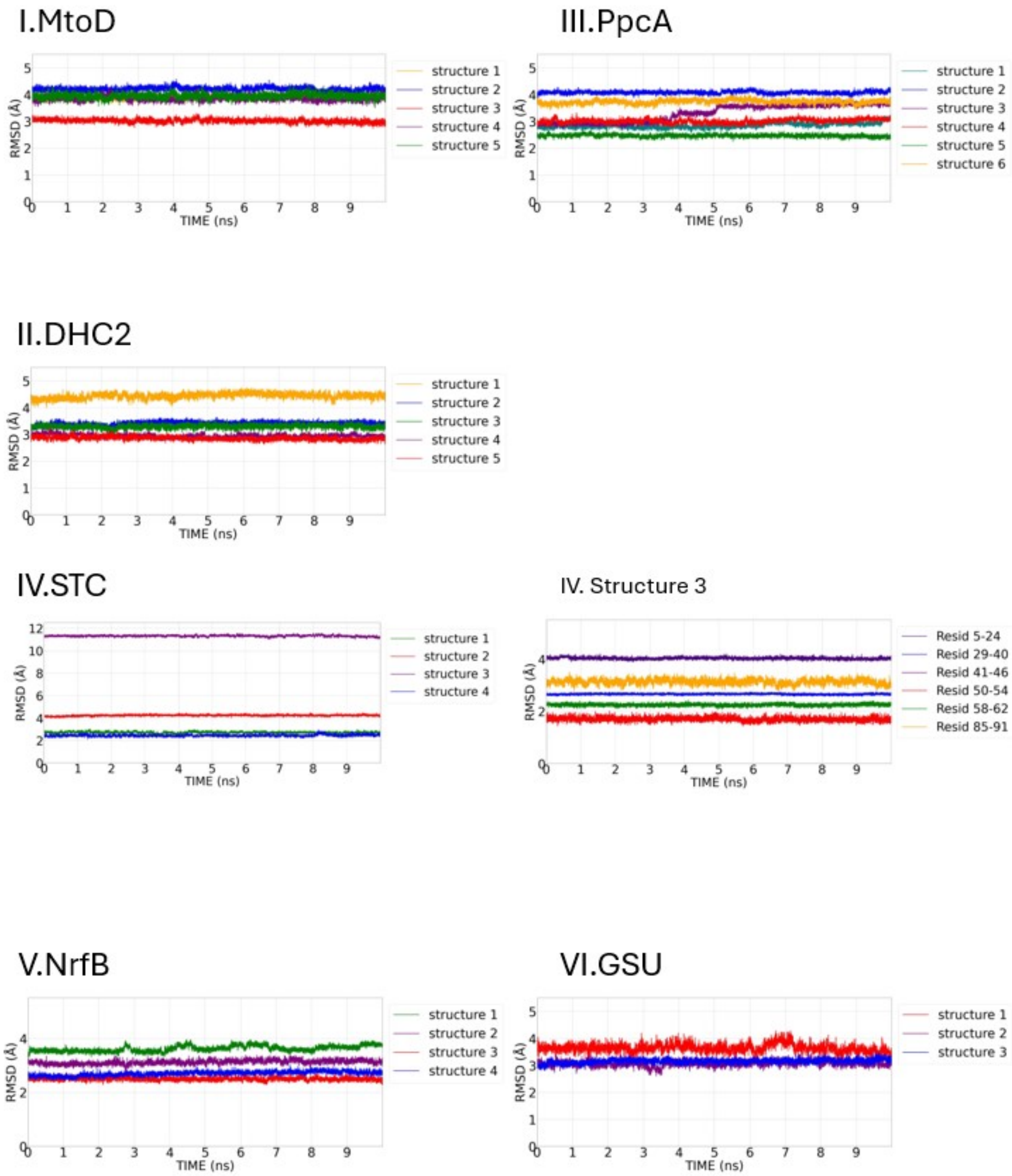

Figure S7: RMSD of protein backbone atoms relative to experimental crystal structure along the last 10 ns MD simulation of the protein junctions. Further breakdown is provided for For STC structure **3** the RMSD of the folded secondary structure elements are displayed on the panel to the right indicating that they remain folded and intact. The large RMSD for this structures is due to the disordered secondary structure elements of the protein that interact with the electrode.

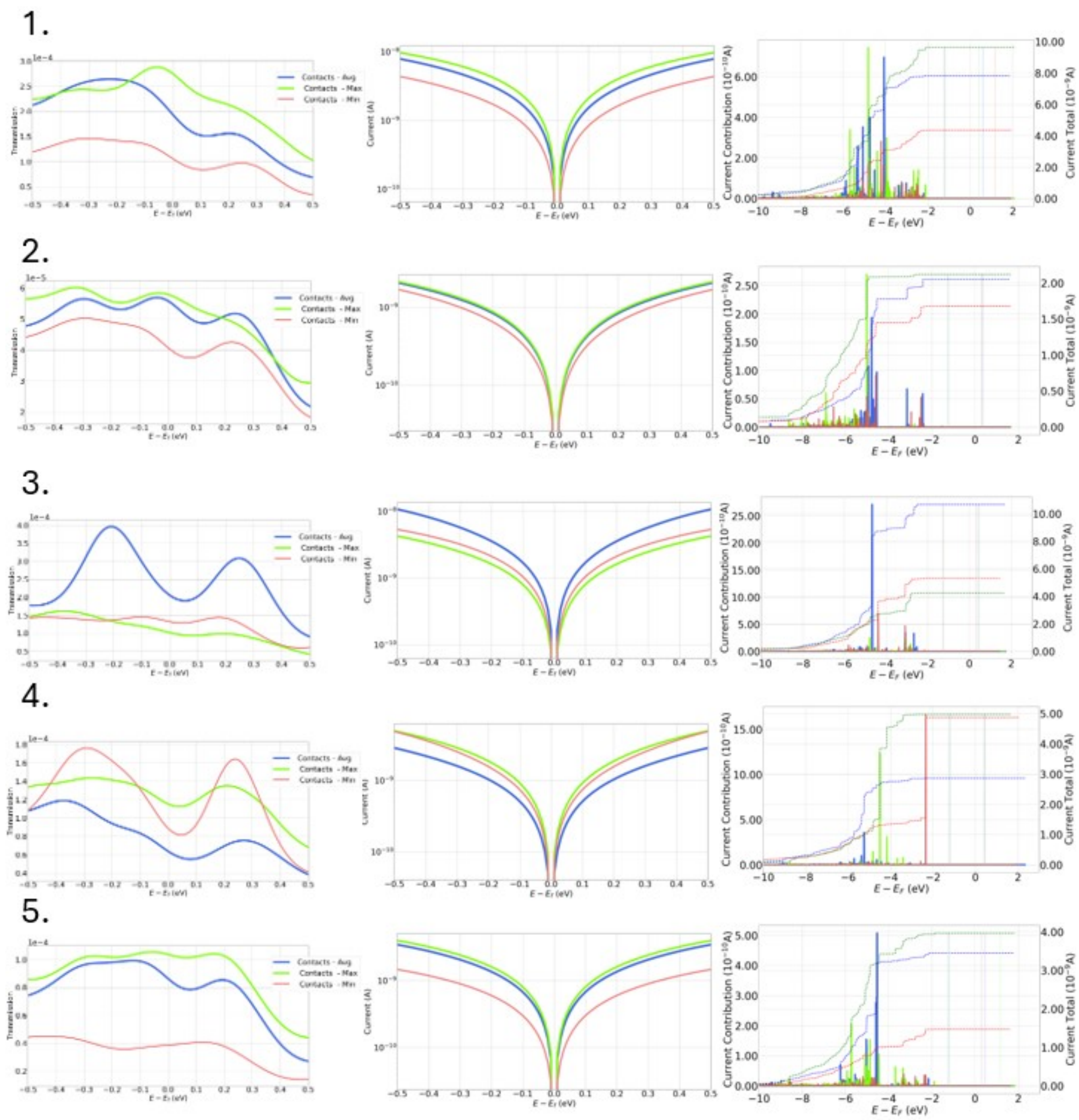

Figure S8: Transmission functions, I-V curves and tunneling current breakdown for all MtoD junctions.

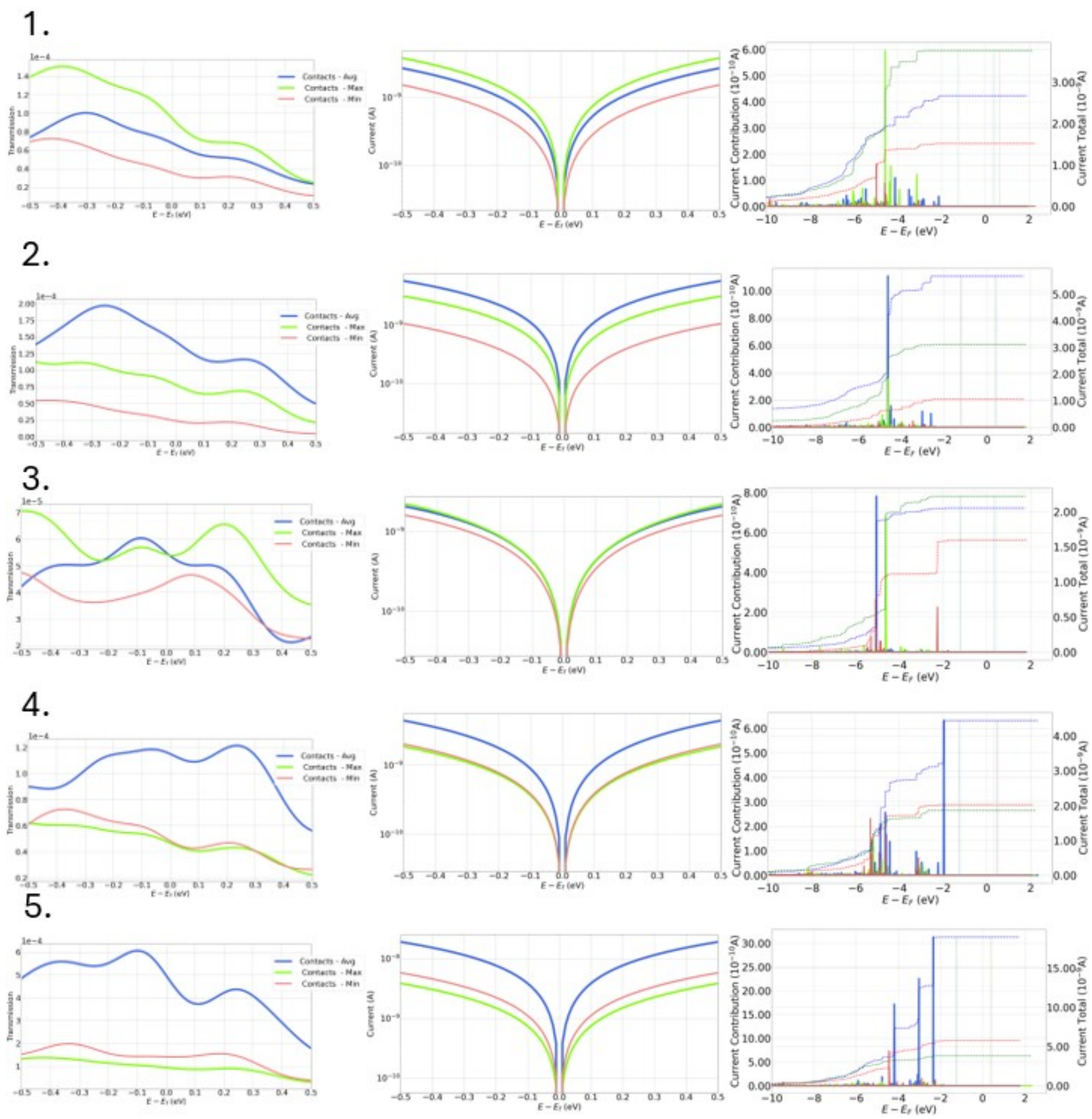

Figure S9: Transmission functions, I-V curves and tunneling current breakdown for all DHC2 junctions.

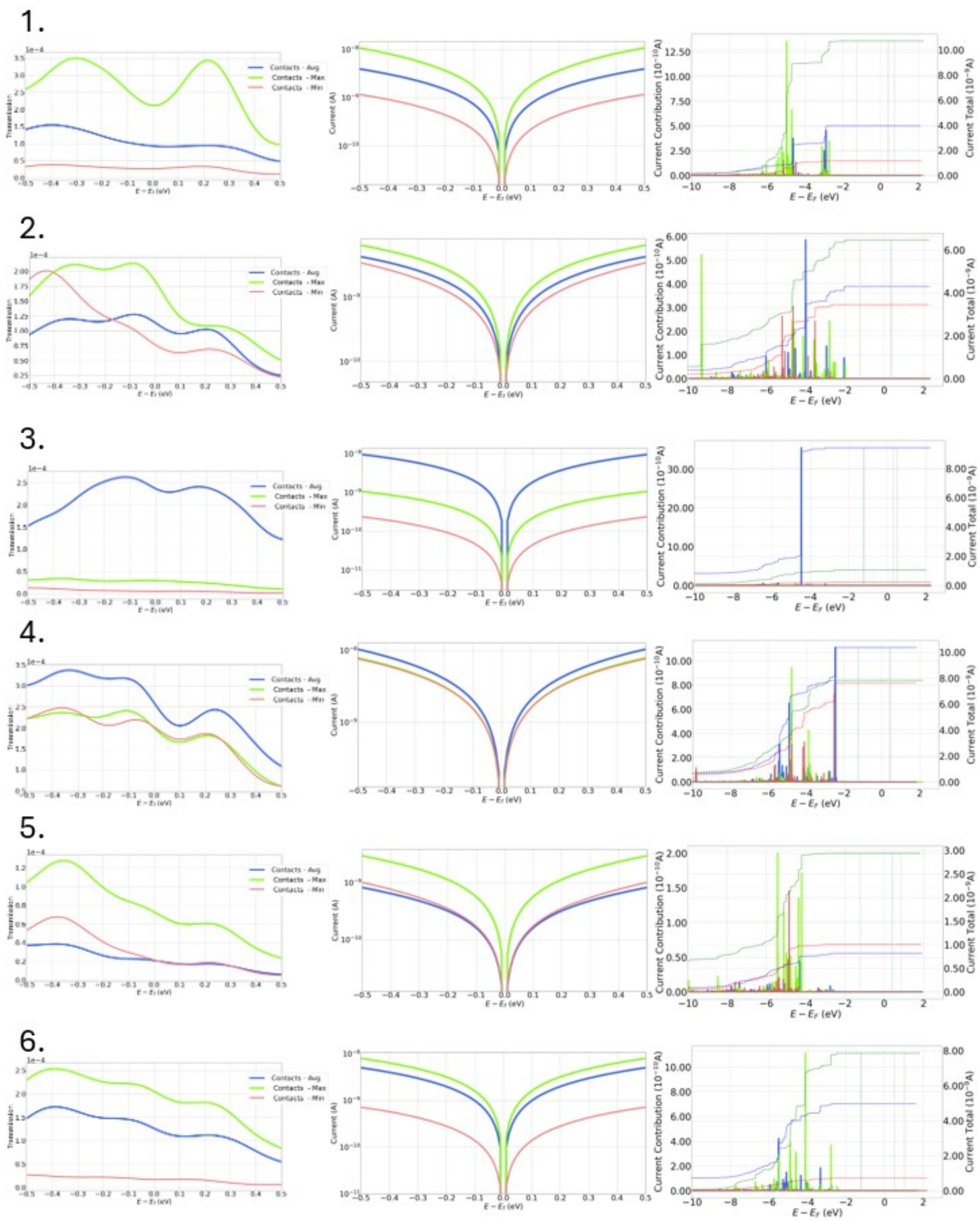

Figure S10: Transmission functions, I-V curves and tunneling current breakdown for all PpcA junctions.

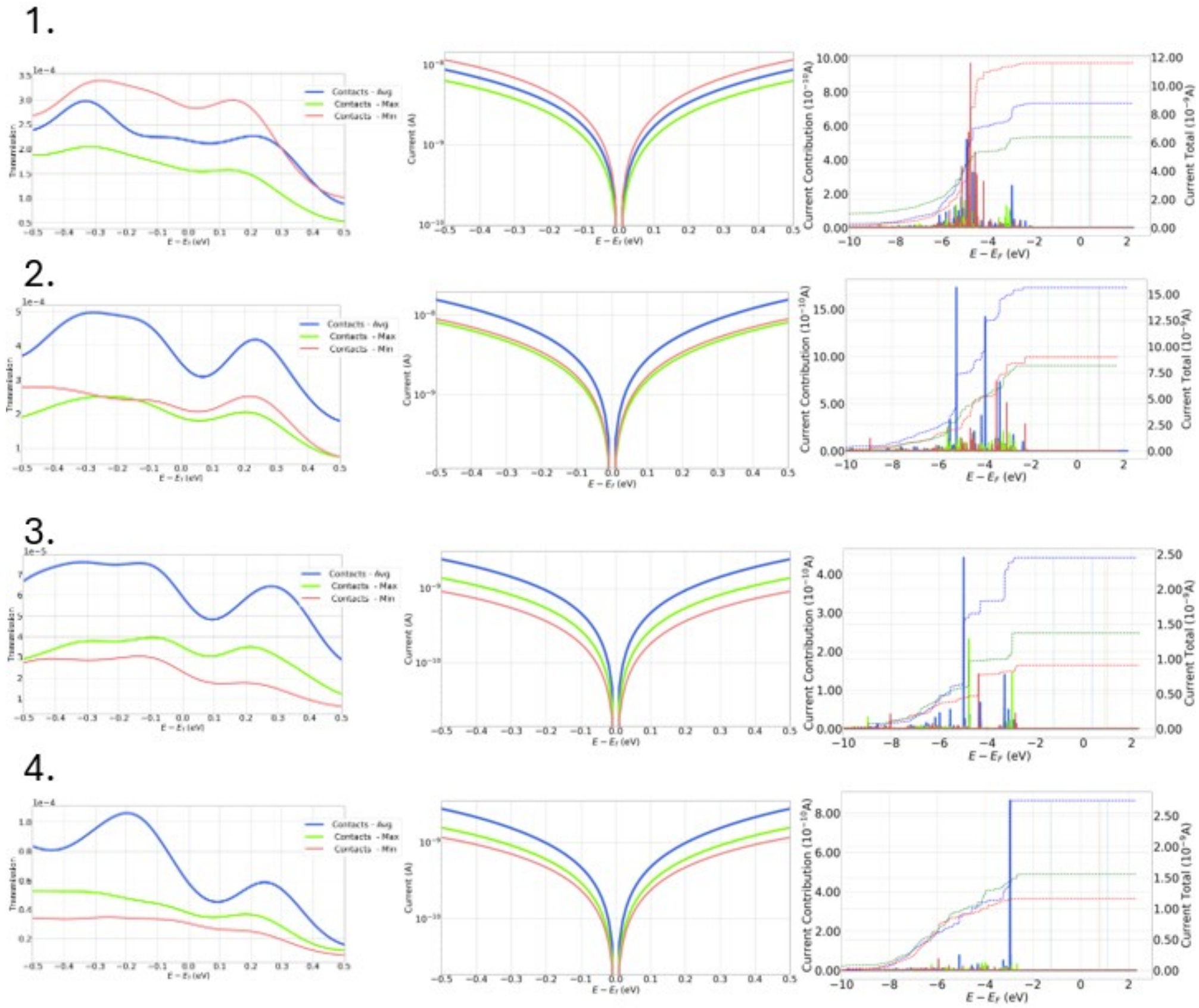

Figure S11: Transmission functions, I-V curves and tunneling current breakdown for all STC junctions.

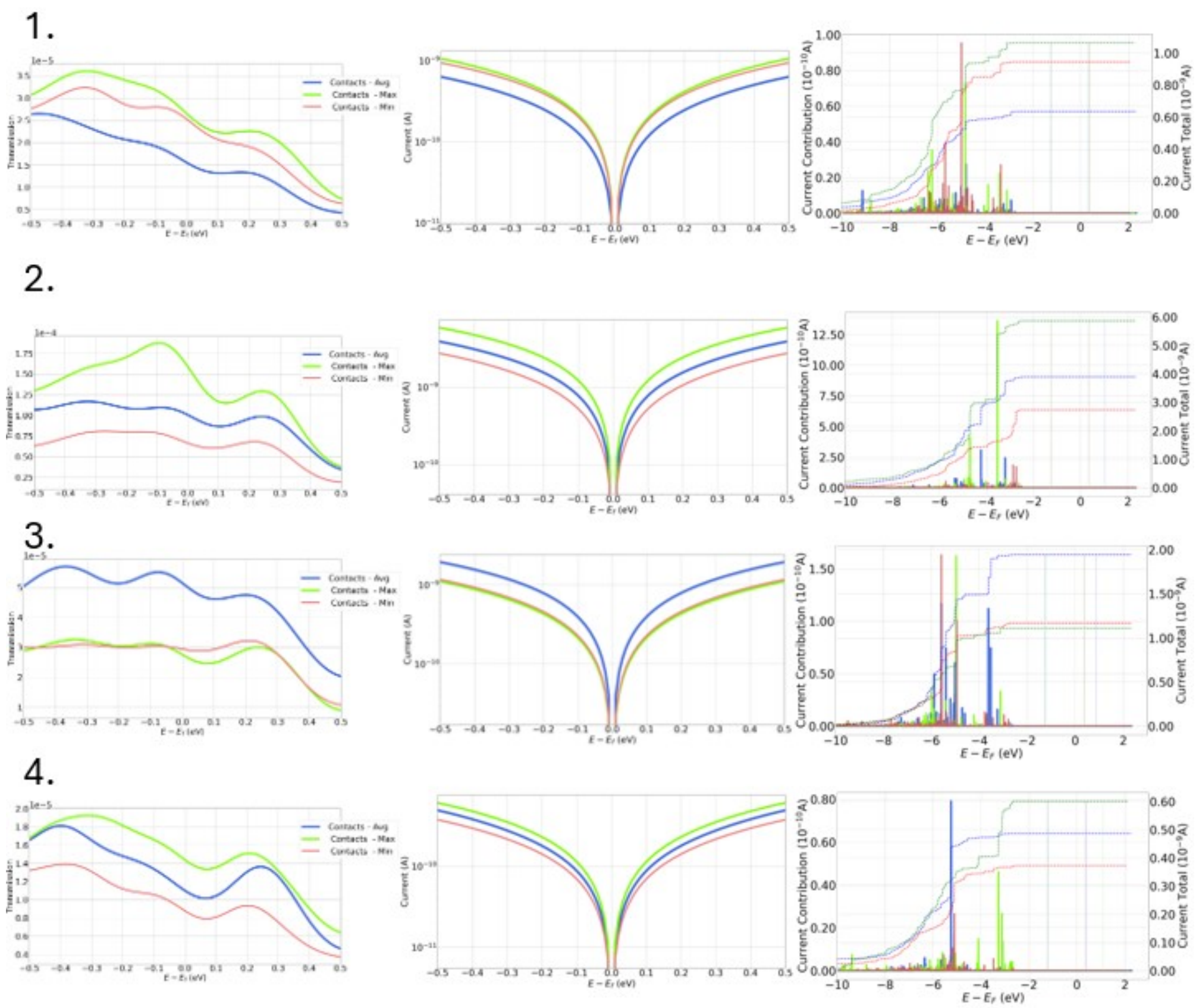

Figure S12: Transmission functions, I-V curves and tunneling current breakdown for all NrfB junctions.

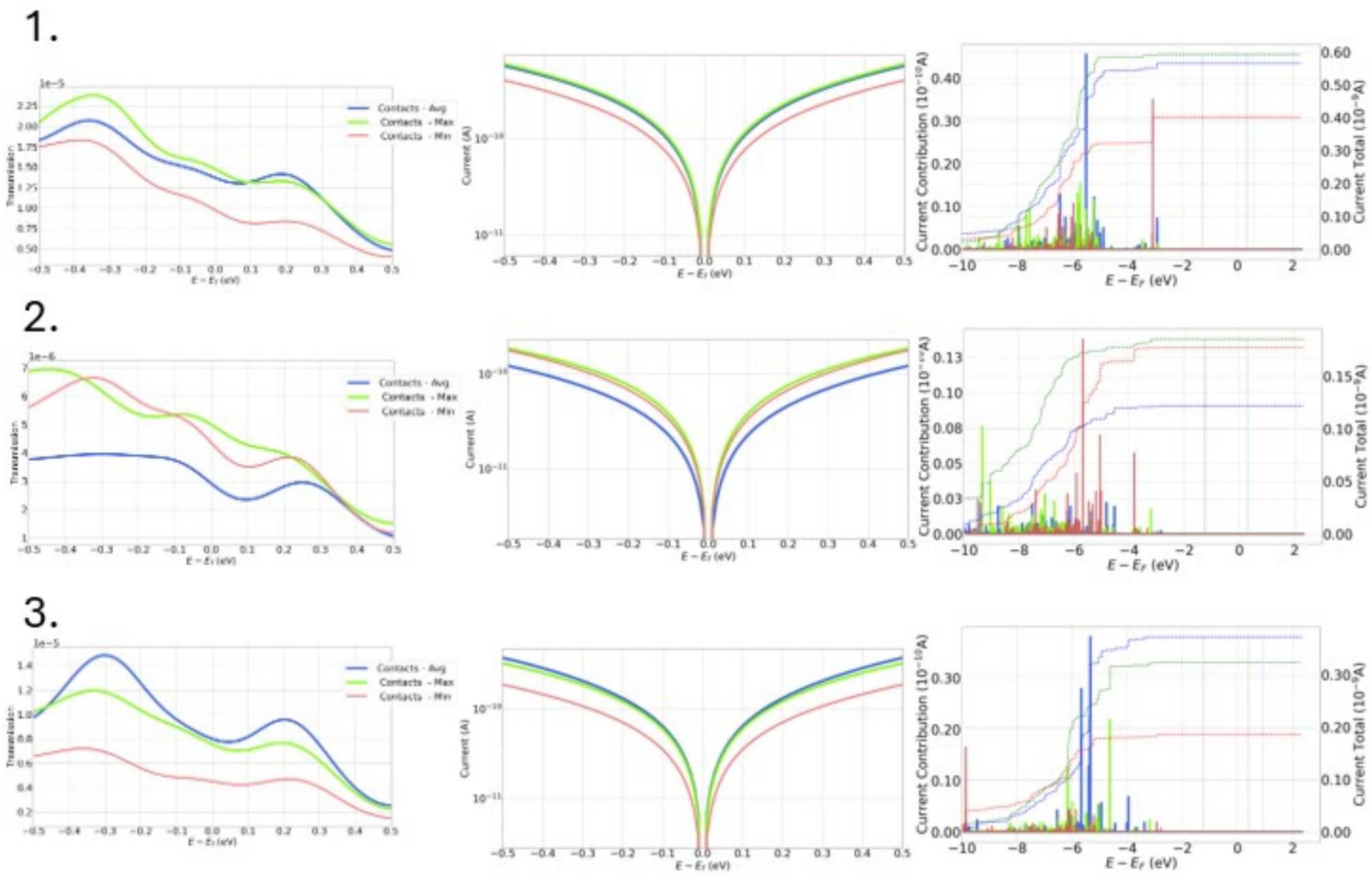

Figure S13: Transmission functions, I-V curves and tunneling current breakdown for all GSU junctions.

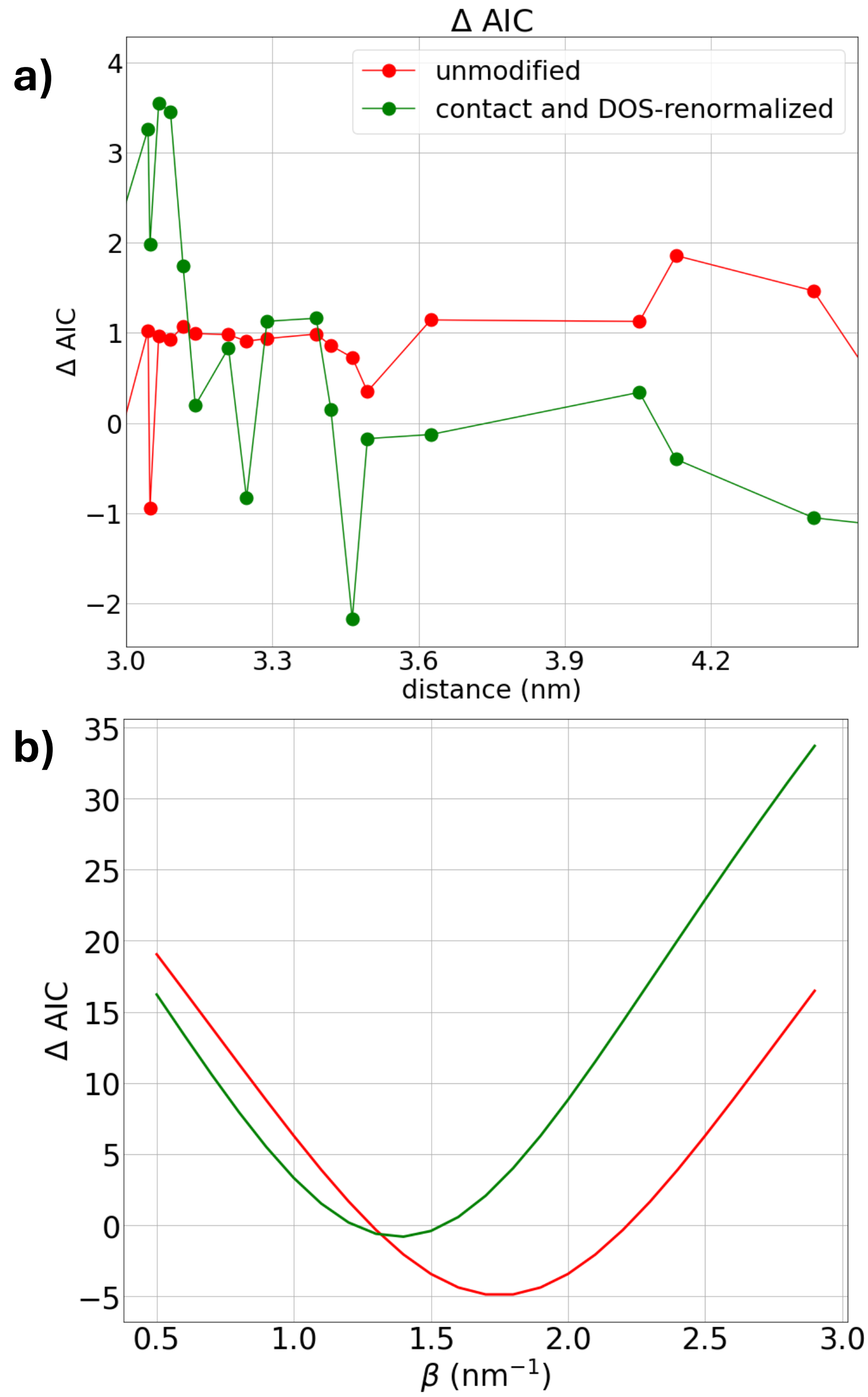

Figure S14: Akaike information criterion (AIC) for the conductance decay models shown in Figure 3 main text. In panel (a) ΔAIC = AIC (2-exponential) − AIC (1-exponential) is shown for different cross-over distances for the 2-exponential model. At each cross-over distance the best 2-exponential and 1-exponential fits are used. For the 1-exponential fit, $\beta = 1.1\,\mathrm{nm}^{-1}$ (unmodified data) and $\beta = 1.3\,\mathrm{nm}^{-1}$ (renormalized data) is independent on the cross-over distance. In panel (b) ΔAIC = AIC (6-intercept) − AIC (1-exponential) is shown for different β values of the 6-intercept model with the β value for the 1-exponential model kept fixed at the optimal value, as in panel (a). Data in red are for the unmodified computed conductances and data in green for the contact and density of states-renormalized computed conductances. Notice that for the best 6-intercept model fit ($\beta = 1.8\,\mathrm{nm}^{-1}$), ΔAIC $= -4.9$ is close to a minimum and the 6-intercept model is statistically preferred over the 1-exponential fit. None of the 2-exponential model fits are statistically preferred over the 1-exponential model fits.

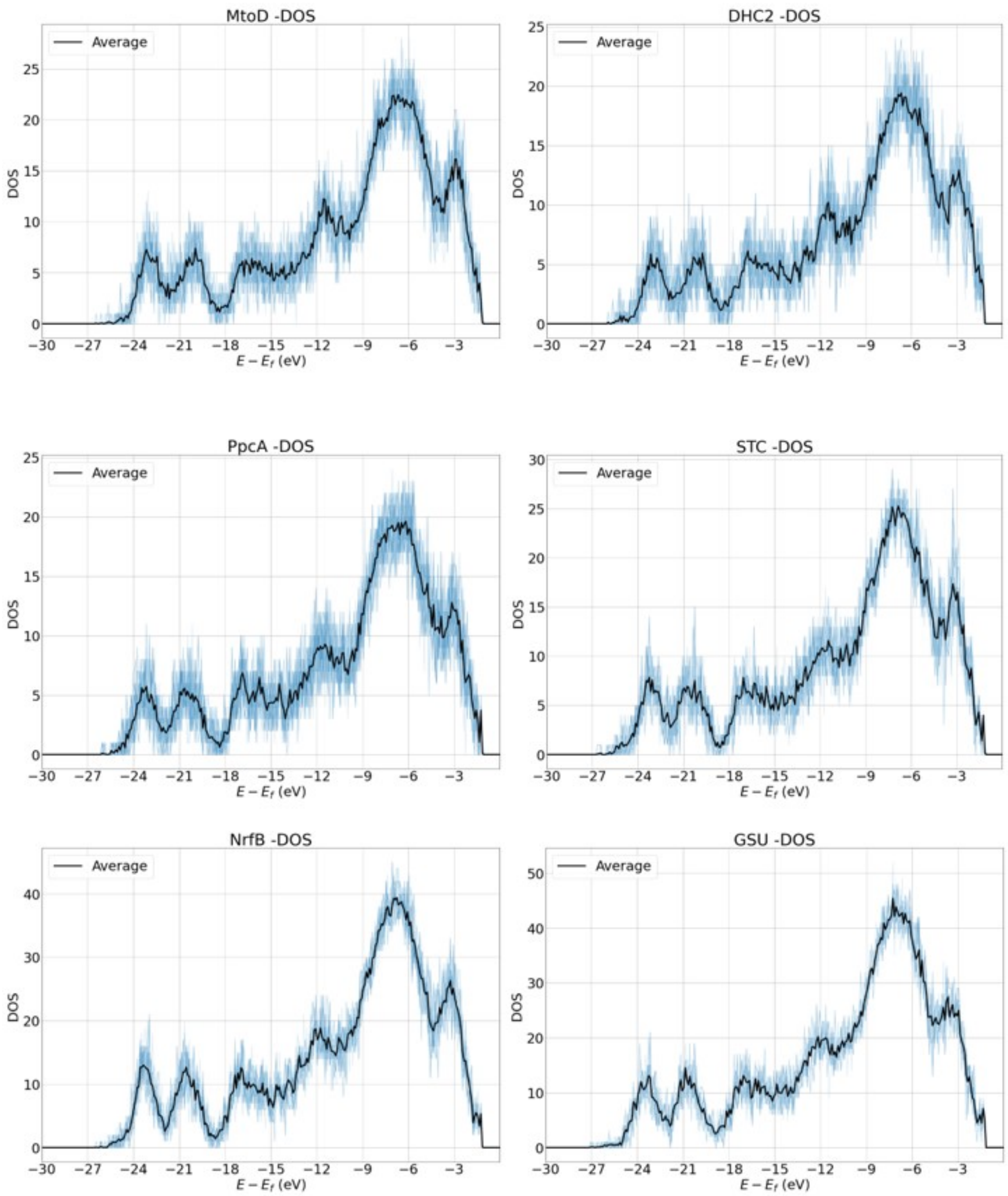

Figure S15: Density of protein electronic states averaged over all structures of a given protein junction, for all proteins.

References for Supporting Information

(1) Wright, L. B.; Rodger, P. M.; Corni, S.; Walsh, T. R. GolP-CHARMM: First-principles based force fields for the interaction of proteins with Au (111) and Au (100). *J. Chem. Theory Comput.* **2013**, *9*, 1616–1630.

(2) Futera, Z.; Wu, X.; Blumberger, J. Tunneling-to-hopping transition in multiheme cytochrome bioelectronic junctions. *J. Phys. Chem. Lett.* **2023**, *14*, 445–452.

(3) Futera, Z.; Blumberger, J. Adsorption of amino acids on gold: Assessing the accuracy of the GolP-CHARMM force field and parametrization of Au–S bonds. *J. Chem. Theory Comput.* **2018**, *15*, 613–624.

(4) Bekker, H.; Berendsen, H.; Dijkstra, E.; Achterop, S.; Vondrumen, R. v.; Vanderspoel, D.; Sijbers, A.; Keegstra, H.; Renardus, M. Gromacs-a parallel computer for molecular-dynamics simulations. 4th international conference on computational physics (PC 92). 1993; pp 252–256.

(5) Van Der Spoel, D.; Lindahl, E.; Hess, B.; Groenhof, G.; Mark, A. E.; Berendsen, H. J. GROMACS: fast, flexible, and free. *J Comput. Chem.* **2005**, *26*, 1701–1718.

(6) Beckwith, C. R.; Edwards, M. J.; Lawes, M.; Shi, L.; Butt, J. N.; Richardson, D. J.; Clarke, T. A. Characterization of MtoD from Sideroxydans lithotrophicus: a cytochrome c electron shuttle used in lithoautotrophic growth. *Front. Microbiol.* **2015**, *6*, 136987.

(7) Heitmann, D.; Einsle, O. Structural and biochemical characterization of DHC2, a novel diheme cytochrome c from Geobacter sulfurreducens. *Biochemistry-US* **2005**, *44*, 12411–12419.

(8) Morgado, L.; Bruix, M.; Pokkuluri, P. R.; Salgueiro, C. A.; Turner, D. L. Redox-

and pH-linked conformational changes in triheme cytochrome PpcA from Geobacter sulfurreducens. *Biochem. J.* **2017**, *474*, 231–246.

(9) Clarke, T. A.; Cole, J. A.; Richardson, D. J.; Hemmings, A. M. The crystal structure of the pentahaem c-type cytochrome NrfB and characterization of its solution-state interaction with the pentahaem nitrite reductase NrfA. *Biochem. J.* **2007**, *406*, 19–30.

(10) Pokkuluri, P.; Londer, Y.; Duke, N.; Pessanha, M.; Yang, X.; Orshonsky, V.; Orshonsky, L.; Erickson, J.; Zagyanskiy, Y.; Salgueiro, C. Structure of a novel dodecaheme cytochrome c from Geobacter sulfurreducens reveals an extended 12 nm protein with interacting hemes. *J. Struct. Biol.* **2011**, *174*, 223–233.

(11) Kokhan, O.; Ponomarenko, N. S.; Pokkuluri, P. R.; Schiffer, M.; Mulfort, K. L.; Tiede, D. M. Bidirectional photoinduced electron transfer in ruthenium (II)-tris-bipyridyl-modified PpcA, a multi-heme c-type cytochrome from Geobacter sulfurreducens. *J. Phys. Chem. B* **2015**, *119*, 7612–7624.

(12) Futera, Z.; Blumberger, J. Electronic couplings for charge transfer across molecule/metal and molecule/semiconductor interfaces: Performance of the projector operator-based diabatization approach. *J. Phys. Chem. C* **2017**, *121*, 19677–19689.

(13) Perdew, J. P.; Burke, K.; Ernzerhof, M. Generalized Gradient Approximation Made Simple. *Phys. Rev. Lett.* **1996**, *77*, 3865–3868.

(14) Goedecker, S.; Teter, M.; Hutter, J. Separable dual-space Gaussian pseudopotentials. *Phys. Rev. B* **1996**, *54*, 1703–1710.

(15) Kuhne, T. D.; Iannuzzi, M.; Ben, M. D.; Rybkin, V. V.; Seewald, P.; Stein, F.; Laino, T.; Khaliullin, R. Z.; Schutt, O.; Schiffmann, F. et al. CP2K: An electronic structure and molecular dynamics software package - Quickstep: Efficient and accurate electronic structure calculations. *J. Chem. Phys.* **2020**, *152*, 194103.

(16) Hutter, J.; Iannuzzi, M.; Schiffmann, F.; VandeVondele, J. CP2K: atomistic simulations of condensed matter systems. *WIRES Comput. Mol. Sci.* **2014**, *4*, 15–25.

(17) Neaton, J. B.; Hybertsen, M. S.; Louie, S. G. Renormalization of Molecular Electronic Levels at Metal-Molecule Interfaces. *Phys. Rev. Lett.* **2006**, *97*, 216405.

(18) Darancet, P.; Widawsky, J. R.; Choi, H. J.; Venkataraman, L.; Neaton, J. B. Quantitative Current-Voltage Characteristics in Molecular Junctions from First Principles. *Nano Lett.* **2012**, *12*, 6250–6254, PMID: 23167709.

(19) Egger, D. A.; Liu, Z.-F.; Neaton, J. B.; Kronik, L. Reliable Energy Level Alignment at Physisorbed Molecule-Metal Interfaces from Density Functional Theory. *Nano Lett.* **2015**, *15*, 2448–2455, PMID: 25741626.

(20) Futera, Z.; Ide, I.; Kayser, B.; Garg, K.; Jiang, X.; Wonderen, J. H. v.; Butt, J. N.; Ishii, H.; Pecht, I.; Sheves, M. et al. Coherent electron transport across a 3 nm bioelectronic junction made of multi-heme proteins. *J. Phys. Chem. Lett.* **2020**, *11*, 9766–9774.
